# Bayesian Integration of Tumor Mutational Signatures and Somatic Features Refines Pathogenicity Assessment of Germline Mismatch Repair Variants

**DOI:** 10.1155/humu/7456837

**Published:** 2026-09-08

**Authors:** Yonatan Amzaleg, Kevin J. McDonnell, Gregory E. Idos, Christina Mathai, Stacy W. Gray, Heather Hampel, David W. Craig

**Affiliations:** ^1^ Department of Integrative Translational Science, Beckman Research Institute, City of Hope, Duarte, California, USA, cityofhope.org; ^2^ Division of Clinical Cancer Genomics, Department of Medical Oncology and Therapeutics Research, City of Hope, Duarte, California, USA, cityofhope.org; ^3^ Department of Population Sciences, City of Hope Comprehensive Cancer Center, Duarte, California, USA, cityofhope.org; ^4^ Enterprise Data Group, City of Hope, Duarte, California, USA, cityofhope.org

## Abstract

Variants of uncertain significance (VUS) in mismatch repair (MMR) genes represent a persistent bottleneck in germline interpretation for Lynch syndrome, creating a critical opportunity to leverage tumor biology to refine pathogenicity assessment. Although tumor features such as microsatellite instability (MSI) and immunohistochemistry (IHC) are routinely evaluated, they are typically interpreted separately from germline classification, and their quantitative contribution within ACMG/AMP frameworks remains poorly defined. We therefore analyzed paired germline and tumor sequencing data from 1110 tumors across 1073 patients with colorectal or endometrial cancer to determine whether mismatch repair–deficient (MMR‐d) mutational signatures can be quantitatively integrated into Bayesian germline variant interpretation. Using COSMIC single‐base substitution signatures, tumors were classified as MMR‐d or MMR proficient, and an empirically derived likelihood ratio (LR) quantified the association between MMR‐d signatures and pathogenic germline MMR variants. The presence of an MMR‐d signature increased the likelihood of an underlying pathogenic germline MMR variant approximately eightfold (LR ≈ 8; log10 LR ≈ 0.90), whereas its absence provided moderate‐to‐strong benign evidence (LR ≈ 0.156; log10 LR ≈ −0.81). Applying this integrative framework to 45 germline MMR VUS, joint modeling of tumor mutational signatures with additional somatic and variant‐level evidence resulted in clinically significant reclassification of 38 (84.4%) variants, including three reclassified as pathogenic or likely pathogenic and 35 as likely benign. A total of 16 downgraded variants were independently downgraded by Invitae. These findings demonstrate that tumor mutational signatures can be formally incorporated into Bayesian germline interpretation, transforming tumor data into quantitative pathogenicity evidence and offering a principled strategy to reduce VUS burden in hereditary cancer genetics.

## 1. Introduction

The increasing availability of paired germline and tumor sequencing in routine oncology practice presents a significant opportunity to refine variant interpretation through integrated analysis [1, 2]. As tumor–normal sequencing becomes more common across cancer centers, clinicians, and laboratories are increasingly confronted with cases in which both inherited and somatic genomic data are available from the same individual. Despite this convergence, germline, and tumor findings are typically interpreted within separate analytic frameworks. This separation is particularly consequential for mismatch repair (MMR) genes, where variants of uncertain significance (VUS) continue to hinder the diagnosis and management of Lynch syndrome [3].

Lynch syndrome accounts for approximately 5%–6% of colorectal cancers (CRCs) and confers substantial lifetime risks of CRC and endometrial cancer (EC) [4–9]. Germline pathogenic variants in *MLH1*, *MSH2*, *MSH6*, or *PMS2* initiate tumorigenesis through loss of MMR function, followed by somatic inactivation of the second allele via mutation, loss of heterozygosity, or *MLH1* promoter hypermethylation [10]. The resulting mismatch repair deficiency (MMR‐d) produces a characteristic tumor phenotype marked by microsatellite instability (MSI) and elevated mutation burden [11–14]. Although this biological cascade tightly links inherited and somatic events, current interpretive frameworks do not fully exploit this connection when classifying germline variants [15–17].

In clinical workflows, MMR‐d is evaluated using immunohistochemistry (IHC) [11, 12], MSI, and, increasingly, tumor mutational burden [14] (TMB) or next‐generation sequencing (NGS)–based assays [18]. These modalities characterize tumor phenotype and guide therapeutic decisions, yet their results are often considered ancillary to germline interpretation. Under current ACMG/AMP guidelines, variant classification relies primarily on categorical evidence types such as population frequency, segregation data, functional assays, and computational prediction [19]. Although tumor features may be considered supportive evidence in selected contexts, they are not routinely incorporated as calibrated, quantitative modifiers of pathogenicity likelihood. Consequently, many MMR gene variants remain classified as VUS even when tumor‐level data suggest functional impairment.

Tumor mutational signature analysis provides a particularly promising bridge between somatic phenotype and inherited mechanism. COSMIC‐defined single‐base substitution (SBS) signatures associated with MMR‐d capture the biochemical imprint of defective DNA repair and reflect the cumulative mutational processes operating within the tumor genome [20–22]. As tumor sequencing becomes increasingly standardized, these signatures are frequently available yet rarely used as formal evidence in germline classification [21, 22]. A key limitation has been the absence of empirically derived likelihood ratios (LR) linking specific mutational signatures to the probability that an underlying germline variant is pathogenic rather than incidental or somatic in origin.

Bayesian reinterpretations of the ACMG/AMP framework offer a principled approach to addressing this limitation. By assigning quantitative LRs to defined evidence categories, Bayesian models enable formal posterior probability calculations and transparent evidence integration [23, 24]. Previous studies have shown the value of multifactorial likelihood models in integrating data from various sources to reclassify VUS [25–29]. Recent efforts have expanded this Bayesian approach to incorporate tumor‐specific and functional features for the MMR gene [7, 30, 31]. However, to our knowledge, no study has systematically calibrated COSMIC‐defined MMR‐d mutational signatures as an independent probabilistic line of evidence within a Bayesian germline interpretation framework, nor has their operating performance been evaluated at scale in a large paired tumor–germline cohort [22].

Here, leveraging access to paired germline and tumor sequencing data from 1110 tumors across 1073 individuals with colorectal or EC, we systematically evaluate the utility of MMR‐d mutational signatures as quantitative evidence for germline MMR variant interpretation. For this study, we will focus on the single‐nucleotide variant (SNV) signatures in which there are over 60 mutational signatures across various cancers, with 96 possible variant types based on nucleotide change patterns [20]. We define true positives, false positives, true negatives, and false negatives classifications to derive an empirical LR linking the presence or absence of MMR‐d signatures to pathogenic germline variants. This calibrated LR is then incorporated into a Bayesian reinterpretation framework alongside additional tumor‐derived features, including second somatic hits in the same gene [32], allele‐specific expression (ASE), and structured interpretation of immunohistochemical patterns.

By applying this integrated model to a curated set of germline MMR VUS, we demonstrate that tumor‐derived molecular data can meaningfully shift posterior probabilities of pathogenicity in both directions, enabling principled upgrades and downgrades of variant classification. Beyond establishing MMR‐d mutational signatures as a quantitative source of evidence for Lynch syndrome–associated genes, our results illustrate a broader paradigm for coordinated tumor–germline inference. As paired sequencing becomes increasingly routine across cancer types, empirically calibrated tumor phenotypes may serve as generalizable probabilistic modifiers within Bayesian variant interpretation, providing a scalable framework for reducing VUS burden in hereditary cancer genetics.

## 2. Materials and Methods

### 2.1. Research Subjects and Samples

Subjects used in this study consented to broad data sharing at the City of Hope Center for Precision Medicine under the INSPIRE (Implementing Next‐generation sequencing for Precision Intervention and Risk Evaluation) project. The study was approved by the City of Hope Institutional Review Board (IRB #24795) and was conducted in accordance with institutional guidelines and the principles outlined in the Declaration of Helsinki. Patient genomic and clinical data were accessed through POSEIDON, a secure, cloud‐based Oncology Insights Engine that enables the exploration, visualization, and analysis of clinical‐genomic data integrated with public datasets [33]. We restricted our analysis to patients with CRC or EC who underwent both germline and tumor sequencing as part of the INSPIRE project. Clinical and sequencing metadata were obtained from POSEIDON and included age at diagnosis, sex, tumor stage, TMB, MSI status, MMR IHC, specimen site, specimen type, and SNOMED disease ontology designations. To minimize potential artifacts arising from low tumor content, we excluded tumors with estimated tumor purity below 15% from the study. Tumors with insufficient content fail to capture the representative somatic mutational landscape and thus underestimate TMB and mutational signatures such as those associated with MMR‐d. The final dataset comprised 1073 unique patients with either CRC or EC, with a total of 1110 samples.

### 2.2. Genomic Data

For each patient in the cohort, we had access to a multigene panel testing performed by Invitae, which consisted of 154 cancer genes and a 34‐gene ACMG secondary findings panel. We received a comprehensive single tab‐delimited Invitae variant file of all the patients′ germline variants identified using that panel. Because the Invitae file originally contained only transcript‐based HGVS identifiers, genomic (NC‐level) coordinates were generated using Mutalyzer v2.0 [34] to standardize variant representation across datasets. This standardization enabled integration with external variant resources, such as ClinVar (Versions 2018–2025) [35], for subsequent tracking of classification changes. We received the FASTQ files for germline and tumor whole exome and tumor transcriptome for all patients; in addition, we received somatic VCFs from Ashion/Exact Sciences based on their whole‐exome sequencing (WES) of patients′ tumor DNA. The WES of germline and tumor DNA FASTQ pairs and tumor RNA FASTQ files were processed as previously described [36]. Genomic HGVS coordinates are reported using GRCh37 reference accessions, whereas whole‐exome alignment and mutational‐signature analysis were performed using the GRCh38 genome build.

### 2.3. Somatic Variant Calling

Somatic variants were identified using five independent somatic variant callers (Mutect2, Strelka2, Octopus, VarDict, and Lancet). For each tumor sample, a multicaller consensus strategy was employed. VCF files from all five callers were merged, and only variants detected by at least three of the five callers were retained, minimizing false positives (FPs). Filtered VCFs were processed using a custom script that implemented the multicaller consensus strategy via the INFO field tag “CC” (caller count). Variants with a CC greater than or equal to 3 were written to a new per‐sample VCF file and served as input for downstream mutational signature analysis.

### 2.4. Mutational Signature Analysis

Mutational signatures were determined using SigProfilerAssignment (v1.0.21) under the COSMIC v3.6 reference framework with the GRCh38 genome build [20]. The analysis was performed in exome mode using 96‐channel SBS (SBS96) contexts. For each tumor specimen, signatures were fitted using the cosmic_fit() function, generating per‐signature activity tables and relative‐contribution plots. The full pipeline was executed within Singularity containers to ensure reproducibility.

An individual COSMIC signature was classified as present when its attributed activity was at least 200 mutations. Samples with activities corresponding to MMR‐d (SBS6, SBS14, SBS15, SBS20, SBS21, SBS26, and SBS44), POLE (SBS10a and SBS10b), POLD1 (SBS10c and SBS10d), HRD (SBS3), MUTYH (SBS18 and SBS36), APOBEC (SBS2 and SBS13), tobacco‐associated (SBS4, SBS29, SBS92, SBS100, and SBS109), or UV‐associated (SBS7a‐d and SBS38) signatures were classified according to their dominant mutational process (Figure S1). Samples exhibiting multiple active processes were assigned combined classifications (e.g., MMR‐d + POLE). They were considered MMR‐d positive for downstream LR analyses when an MMR‐d signature was present.

To evaluate the impact of capture footprint on mutational signature detection, a parallel analysis was performed using variants restricted to the FoundationOne CDx 324‐gene panel. Panel‐filtered variants were reanalyzed using SigProfilerAssignment and a panel‐renormalized COSMIC v3.6 reference catalogue generated by rescaling each SBS channel according to the FoundationOne CDx trinucleotide composition before signature fitting. Because the exome‐derived ≥ 200 mutation activity threshold was not directly transferable to panel‐restricted data, panel MMR‐d status was defined using the summed activity of MMR‐d‐associated signatures (SBS6, SBS14, SBS15, SBS20, SBS21, SBS26, and SBS44). Thresholds were evaluated across a range of activity cutoffs, and operating points were selected using either the maximum Youden′s J statistic [37] or a matched‐LR+ approach.

As a complementary analysis, TMB was evaluated as a predictor of pathogenic germline MMR status. Tumors classified as intermediate or high TMB (≥ 10 mut/Mb) were considered TMB‐positive, whereas low‐TMB tumors were considered TMB‐negative. Sensitivity, specificity, positive likelihood ratio (LR+), and negative likelihood ratio (LR−) were calculated using the same germline comparator framework as in the mutational signature analysis.

### 2.5. Bayesian Likelihood Framework

To quantify the evidentiary strength of MMR‐d signatures for classifying variants, we applied a Bayesian LR framework. Tumors were first dichotomized as MMR‐d positive or MMR‐d negative. The respective patients were then assessed for a pathogenic germline mutation in one or more of the canonical MMR genes (*MLH1*, *MSH2*, *MSH6*, and *PMS2*). Patients with a germline MMR VUS but no pathogenic or likely pathogenic germline MMR variant were excluded from LR calibration because their true germline pathogenicity status was unresolved. Patients carrying both a pathogenic germline MMR variant and an MMR VUS were retained in the pathogenic germline group. We then constructed a 2 × 2 contingency table of true positives (TPs), FPs, true negatives (TN), false negatives (FN), where

• TP is a pathogenic germline variant in one of the MMR genes + MMR‐d signature present in tumor.
• FN is a pathogenic germline variant in one of the MMR genes + MMR‐d signature absent in tumor.
• TN is a no pathogenic germline variant in one of the MMR genes + MMR‐d signature absent in tumor.
• FP is a no pathogenic germline variant in one of the MMR genes + MMR‐d signature present in tumor.

From these, the sensitivity and specificity values were calculated as follows:

• Sensitivity = TP/(TP + FN) and specificity = TN/(TN + FP).

The LR+ was then computed as

• LR + = sensitivity/(1 − specificity).
• LR − = (1 − sensitivity)/specificity.

The log10(LR+) was used to interpret the evidence strength according to the ACMG/AMP Bayesian guidelines (supporting = 0.32, moderate = 0.64, strong = 1.27, very strong = 2.54) [23]. To visualize how the observed LR+ would influence variant interpretations under different prior probabilities, we generated a posterior probability curve across priors ranging from 0.01 (meaning there is a 1% prior probability that a germline VUS in an MMR gene is pathogenic) to 0.80 (there is a 80% prior probability that a germline VUS in an MMR gene is pathogenic) using the formula (Figure S2A).

(1) Pposterior=LR×Pprior1−Pprior+LR×Pprior 

Where **P**_**posterior**_ is the posterior probability that the variant is pathogenic; **P**_**prior**_ is the prior probability that the variant is pathogenic; and **LR** is the LR associated with a single piece of evidence (in this case, the presence or absence of MMR‐d signature). Plots were produced in Python (v3.8) using Matplotlib and Seaborn.

### 2.6. Posterior Probability Reclassification of Germline VUS Using a Bayesian Framework

**Figure 1:**
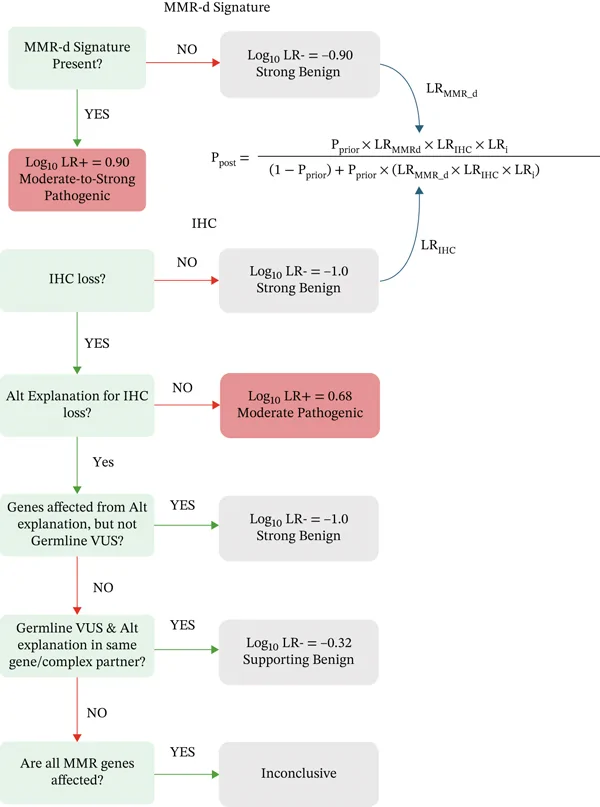
Bayesian integration of somatic mutational signatures and IHC features as additive evidence to refine germline MMR VUS pathogenicity. This schematic illustrates how independent tumor‐derived evidence streams are converted into likelihood ratios (LRs) and combined to update a germline variant′s probability of pathogenicity. The MMR‐d signature track assigns an LR supporting pathogenicity when an MMR‐deficient mutational signature is present (example shown as log_10_(LR+) = 0.91) and assigns an LR supporting benignity when the signature is absent (example shown as log_10_(LR−) = −0.81). The IHC track assigns LRs based on the presence or absence of MMR protein loss and the interpretability of that loss: intact IHC (no loss) provides strong benign evidence (example log_10_(LR−) = −1.0), whereas IHC loss without a supported alternative explanation provides pathogenic evidence (example log_10_(LR+) = 0.68). When an alternative explanation plausibly accounts for the observed IHC pattern, benign evidence is applied (example log10(LR−) = −1.0). When the germline VUS and alternative explanation involve the same gene or obligate partner relationships, the benign evidence is reduced (example log_10_(LR−) = −0.32, supporting benign). Cases in which all core MMR proteins appear affected without a coherent mechanistic explanation are labeled inconclusive. The central equation summarizes evidence aggregation: the prior probability of pathogenicity (*P*_prior_) is updated by multiplying evidence‐specific LRs from the MMR‐d signature (*L* *R*_MMR−d_), IHC‐based features (*L* *R*_IHC_), and any additional evidence terms (*L* *R*_*i*_) to yield the posterior probability (*P*_post_). Arrows indicate that evidence from each modality contributes to the final posterior, rather than representing mutually exclusive decision branches.

To further examine the classification of VUS canonical MMR genes, we employed a Bayesian variant‐level framework to compute variant‐specific posterior probabilities based on available molecular and genomic features. For each VUS case, the following features were considered as contributing evidence toward pathogenicity: (1) presence of an MMR‐d mutational signature in the tumor, (2) presence of a somatic second hit in the same gene as the germline VUS, and (3) statistically significantly reduced mutant ASE in tumor RNA relative to tumor DNA at the variant site. Conversely, features contributing toward benign classification included: (1) absence of an MMR‐d signature, (2) somatic hits in different MMR genes, (3) mutant ASE in the wild‐type direction, (4) *MLH1* promoter hypermethylation, and (5) discordant IHC (e.g., loss of *MSH6/MSH2* protein in a patient with a germline *PMS2* VUS [38, 39]) (Figure 1, Table 1). Its evidentiary strength is aligned with the ACMG/AMP Bayesian point‐based system [23]. Tumor‐derived features were not assumed to be universally independent. When multiple findings reflected a shared underlying biological mechanism, evidence was interpreted conservatively and was not multiplied mechanically. Gene concordance, alternative molecular explanations, and potential dependence among IHC, somatic second hits, MSI, TMB, and mutational signatures were considered during variant‐level curation. The LR for MMR‐d signature (approximately 8.0) was derived from the cohort‐wide analysis described above, and second hits and mutant ASE were assigned moderate‐strength and supporting‐strength LRs (e.g., LR = 4.3 and 2.1, respectively) based on literature‐derived estimates and biological plausibility [23, 24, 41, 42]. Conversely, benign‐associated features were assigned inverse LRs. For each variant, the final posterior probability was computed using the formula:

(2) Pposterior=LR1×LR2×⋯×LRn×Pprior LR1×LR2×⋯×LRn×Pprior+1−Pprior 

**Table 1:** Bayesian evidence categories and likelihood ratio assignments used for germline MMR variant classification.

Category	Description	Example Scenario	Interpretation	ACMG Strength	Log10(LR)	LR+/LR−
1. Strong benign	Alternative explanation with matching IHC, not involving VUS gene	*MLH1* hypermethylation + IHC *MLH1*/*PMS2* loss + VUS in *MSH6*	VUS is unlikely to be causal	Strong benign	−1.0	0.1
2. Moderate benign	Alternative explanation with IHC loss in MMR partner gene of VUS	*MLH1* hypermethylation + *PMS2* VUS + *MLH1*/*PMS2* loss	Partner gene loss may account for IHC, benign leaning	Moderate benign	−0.68	0.21
3. Supporting benign	Alternative explanation but IHC loss includes VUS gene (ambiguous)	*MLH1* methylation + *MSH6* VUS + *MLH1*/*MSH6* loss	Unclear if VUS gene loss is primary or collateral	Supporting benign	−0.32	0.48
4. Supporting pathogenic	IHC loss matches VUS gene, no alternative explanation	*MSH6* VUS + IHC *MSH6* loss (no *MLH1* methylation or 2nd hit elsewhere)	Suggests VUS drives phenotype	Supporting pathogenic	0.32	2.1
5. Moderate pathogenic	VUS gene shows biallelic inactivation (germline + somatic LOF), IHC loss matches	*MSH6* VUS + somatic frameshift in *MSH6* + *MSH6* loss	Strong mechanistic link to tumor phenotype	Moderate pathogenic	0.63	4.3
6. Supporting pathogenic	VUS shows mutant Allele‐specific expression	At locus of VUS binomial analysis reveals the mutant is statistically significantly expressed more in tumor RNA than DNA	Suggests VUS drives phenotype	Supporting pathogenic	0.32	2.1
7. Functional	In vitro evidence shows functional impairment in relevant assay	*MSH6* VUS with MMR‐d in cell model	Mechanistically damaging	Supporting pathogenic	0.32	2.1
8. Supporting observational	CRC patient with VUS + matching MMR‐d signature	*MSH6* VUS + Signature present	Correlates with MMR‐d tumor biology	Moderate‐to‐strong pathogenic	0.9	8
9. Population	Absent from population databases	Not found in gnomAD	Rare variant	Moderate pathogenic (PM2_mod)	0.63	4.3
9. Population	Present in population databases at clip of less than 0.002%	If *MLH1* variant is found in 0.0001% in gnomad	Rare variant	Supporting pathogenic (PM2_supp)	0.32	2.1
10. In silico	Damaging by MMR‐specific prediction algorithm (e.g., MAPP‐MMR)	Predicted deleterious	Computational support	Supporting pathogenic (PP3)	0.32	2.1
11. Variant found in disease patients	Variant seen in patients with same phenotype	Variant found in multiple Lynch syndrome patients without confounders	Suggests variant could be meaningful for Lynch Syndrome	Supporting pathogenic (PS4)	0.32	2.1

*Note:* This table summarizes the somatic and variant‐level evidence categories incorporated into the Bayesian framework, with corresponding ACMG/AMP evidence strength assignments, log_10_ likelihood ratios (LR), and linear LRs. Evidence categories include alternative explanations for IHC loss, patterns of MMR‐d mutational signatures, allele‐specific expression, functional assay results, population frequency data, in silico predictions, and case‐level enrichment. LRs were applied directionally to update posterior probabilities for germline MMR variants, consistent with the Bayesian adaptation of ACMG/AMP guidelines. These weights were used for all posterior probability calculations reported in Tables 2, 3, and 4 and associated figures. Evidence strengths were assigned according to the Bayesian reinterpretation of ACMG/AMP criteria, in which moderate evidence corresponds to LRs near 4.3 and supporting evidence corresponds to LRs near 2.1, consistent with established variant classification frameworks and ClinGen recommendations [19, 23, 40].

**Figure 2:**
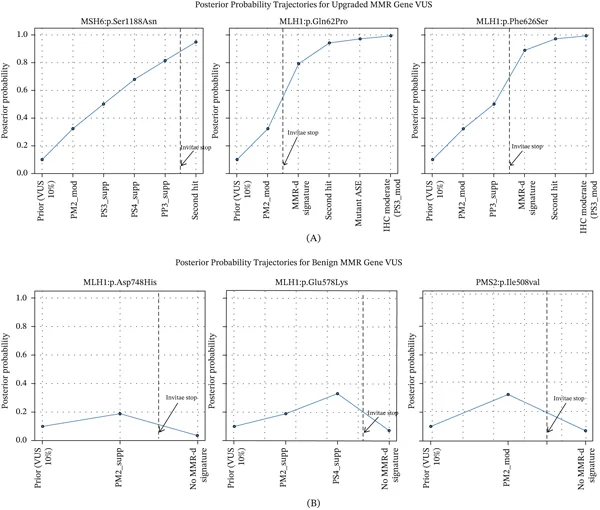
Posterior probability trajectories for upgraded and downgraded germline MMR gene VUS using an integrative Bayesian framework. (A) Upgraded MMR gene VUS (likely pathogenic) posterior probability trajectories for three germline MMR variants that were reclassified as pathogenic or likely pathogenic after incorporation of somatic evidence. Each line represents the cumulative Bayesian updating of posterior probability as successive evidence types are added: population rarity (PM2), in silico predictions (PP3), case‐level or functional evidence (PS3/PS4), and finally somatic tumor features including second somatic hits, mutant allele–specific expression, and the presence of an MMR‐deficient (MMR‐d) mutational signature. The vertical dashed line (“Invitae Stop”) marks the final probability point supported by evidence available to the clinical laboratory (Invitae) at the time of testing. All subsequent increases arise exclusively from tumor‐derived somatic data contributed in this study. For each of the three variants, *MSH6*:p.Ser1188Asn, *MLH1*:p.Gln62Pro, and *MLH1*:p.Phe626Ser, the addition of MMR‐d signature and/or other somatic modalities increased the posterior probability above the 0.90 threshold for likely pathogenic. (B) Benign VUS: Trajectories for variants downgraded following somatic evidence. Posterior trajectories are shown for *MLH1*:p.Asp748His, *MLH1*:p.Glu578Lys, and *PMS2*:p.Ile508Val, three representative variants from the set of 35 VUS downgraded after including somatic evidence. Early increases in posterior probability reflect variant‐level evidence available to Invitae (e.g., PM2_supporting, PS4_supporting). The vertical dashed line (“Invitae Stop”) again marks the boundary of laboratory‐reported evidence. For all three variants, incorporation of the absence of an MMR‐d mutational signature (LR^+^ ≈ 0.156) produced a sharp reduction in posterior probability, placing each variant well below the 10% threshold and consistent with a likely benign interpretation. These examples highlight how tumor‐integrated Bayesian analysis prevents misclassification by distinguishing variants enriched in MMR‐proficient tumors from those enriched in MMR‐deficient tumors.

Where **P**_**posterior**_ is the posterior probability that the variant is pathogenic; **P**_**prior**_ is the prior probability that the variant is pathogenic; and **LR**_***n***_ represents the LR assigned to the *n*th evidence type. Evidence types are explained in Table 1, where the prior probability of a germline VUS being pathogenic was set at 10% and further adjusted based on evidence for or against pathogenicity. These calculations were applied to (a) VUS cases with MMR‐d signatures to assess reclassification to likely pathogenic and (b) VUS cases lacking MMR‐d signatures to assess down‐classification to likely benign. The results were visualized via variant‐specific posterior probability bar plots, stratified by contributing evidence, to illustrate how the addition or absence of MMR‐d and other molecular features shifted interpretation across the ACMG threshold boundaries (pathogenic ≥ 0.99, likely pathogenic 0.90 to < 0.99, VUS 0.10 to < 0.90, and likely benign < 0.10) (Figure 2A,B). We computed 95% confidence intervals (CIs) for sensitivity and specificity using exact binomial (Clopper–Pearson) intervals [43]. LR CIs were calculated on the log scale using standard errors derived from the 2 × 2 table, and 95% CIs were obtained as previously described [44].

### 2.7. Mutant Allele Expression Analysis and Visualization

To assess ASE of candidate germline VUS, we calculated the ratio of reads of mutant versus wild‐type alleles in matched tumor DNA and tumor RNA sequencing data. For each variant, we used pysam (Version 0.23.3) [45] to extract read counts supporting the reference and alternate alleles from the BAM files of the respective patient′s tumor DNA and RNA. A binomial test was then applied to determine whether the observed RNA allele fraction (AF) significantly deviated from the expected 0.5 ratio, using the tumor DNA AF as the baseline expectation. Statistical significance was defined as *p* < 0.05 after multiple testing correction.

Variants showing robust mutant allele expression (RNA AF ≥ 0.20 and statistically significant deviation from the wild‐type) were flagged for further interpretation as supporting evidence for pathogenicity (corresponding to an LR+ of 2.1). In contrast, variants with minimal or absent ASE were considered less likely to be functionally impactful. ASE was treated as supporting evidence because of its greater susceptibility to technical and biological confounders (e.g., tumor purity, copy‐number effects, allele‐mapping bias, and transcript‐level regulation).

To confirm variant position, zygosity, and exclude mapping artifacts, all germline and somatic variants analyzed were manually reviewed using the Integrative Genomics Viewer (IGV) [46]. Tumor and germline BAM files were loaded, and reads were visually inspected at the variant site across both DNA and RNA tracks.

## 3. Results

### 3.1. Overview of Colorectal and EC Cohorts

The cohort was derived from the INSPIRE precision oncology initiative at City of Hope. We restricted the analysis to patients with CRC or EC who underwent paired germline and tumor sequencing. For each patient, we had access to germline multigene panel testing (Invitae), somatic VCFs from Ashion/Exact Sciences, germline and tumor WES, tumor RNA sequencing, and integrated clinical metadata, including age at diagnosis, stage, MSI status, selected IHC data, and TMB.

A total of 1110 tumor samples from 1073 unique patients were included in this study, comprising 804 CRC samples from 772 patients and 306 EC samples from 301 patients. Across the full cohort, the median age at diagnosis was 58 years (IQR 49–66), and 279 patients (26.0%) were classified as “early‐onset”, defined as diagnosis before age 50. The cohort included more female than male patients (701 vs. 372), reflecting the EC population.

Among the 1073 patients, 35 (3.3%) carried at least one pathogenic or likely pathogenic germline MMR variant, corresponding to 36 tumor specimens, as one patient had two tumors sequenced. An additional 44 patients (4.1%) harbored at least one germline MMR VUS, corresponding to 46 tumor specimens. One patient with a pathogenic *PMS2* variant also carried a germline *MLH1* VUS; this patient was retained in the pathogenic group and excluded from the VUS‐only analysis (Table 2, Patient 12). After accounting for this overlap, the VUS‐only cohort comprised 45 distinct germline MMR VUS from 43 patients represented across 45 tumor specimens. The remaining 995 patients had no identified germline MMR variant. For LR calibration, the 45 VUS‐only tumor specimens were excluded because their germline pathogenicity status was unresolved, leaving 1065 evaluable tumor specimens. These germline‐defined groups formed the basis for subsequent comparative analyses of somatic mutational signatures, MSI status, IHC results, and tumor genomic features.

**Table 2:** Clinicopathologic and somatic features of patients with pathogenic germline MMR variants.

ID	Cancer	Signature	Diag Call	Gene	Invitae Sig	HGVS	TMB score	MSI
Patient 1	CRC	MMR‐d + other	TP	*MLH1*	P	Exons 2‐3 deletion	59 mut/Mb	High
Patient 2	CRC	MMR‐d	TP	*MLH1*	P	Exons 6‐12 gain	52 mut/Mb	High
Patient 3	CRC	MMR‐d	TP	*MLH1*	LP	c.2246T>C (p.Leu749Pro)	20 mut/Mb	High
Patient 4	EC	MMR‐d	TP	*MLH1*	P	c.210_213del	21 mut/Mb	High
Patient 5	CRC	None	FN	*PMS2*	P	c.989‐1_1144+2del	2 mut/Mb	Stable
Patient 6	CRC	MMR‐d	TP	*MLH1*	P	c.1381A>T (p.Lys461Ter)	28 mut/Mb	High
Patient 7	EC	MMR‐d	TP	*MSH6*	P	c.1628_1629del (p.Lys543fs)	8 mut/Mb	Stable
Patient 8	CRC	MMR‐d	TP	*PMS2*	P	Exon 12 deletion	32 mut/Mb	High
Patient 9	EC	MMR‐d and POLE	TP	*MSH6*	LP	c.2342C>T (p.Pro781Leu)	237 mut/Mb	High
Patient 10	CRC	MMR‐d and *POLD1*	TP	*MSH2*	LP	c.488T>A (p.Val163Asp)	53 mut/Mb	High
Patient 10	CRC	MMR‐d and *POLD1*	TP	*MSH2*	LP	c.488T>A (p.Val163Asp)	44 mut/Mb	High
Patient 11	CRC	MMR‐d	TP	*MSH2*	P	c.2046_2047del (p.Val684fs)	43 mut/Mb	High
Patient 12	EC	MMR‐d	TP	*PMS2*	P	c.1936del (p.Arg646fs)	14 mut/Mb	High
Patient 13	EC	None	FN	*PMS2*	P	c.1123C>T (p.Gln375Ter)	11 mut/Mb	High
Patient 14	CRC	MMR‐d	TP	*MLH1*	P	c.131_132delinsTT (p.Ser44Phe)	24 mut/Mb	High
Patient 15	CRC	MMR‐d and *POLD1*	TP	*PMS2*	P	Exon 12 deletion	31 mut/Mb	High
Patient 16	CRC	MMR‐d	TP	*MLH1*	P	c.94del (p.Ile32fs)	52 mut/Mb	High
Patient 17	CRC	MMR‐d	TP	*PMS2*	LP	c.2095G>C (p.Asp699His)	16 mut/Mb	High
Patient 18	CRC	MMR‐d	TP	*PMS2*	P	c.251‐2A>T	31 mut/Mb	High
Patient 19	CRC	None	FN	*MSH6*	P	c.3226C>T (p.Arg1076Cys)	2 mut/Mb	Stable
Patient 20	EC	None	FN	*MSH2*	P	c.2038C>T (p.Arg680Ter)	26 mut/Mb	High
Patient 21	EC	MMR‐d	TP	*MSH2*	P	c.1906G>C (p.Ala636Pro)	28 mut/Mb	High
Patient 22	CRC	MMR‐d and POLD1	TP	*MLH1*	P	c.210AGA[1] (p.Glu71del)	44 mut/Mb	High
Patient 23	CRC	None	FN	*MSH6*	P	c.1421_1422dup (p.Gln475fs)	45 mut/Mb	High
Patient 24	CRC	MMR‐d and *POLD1*	TP	*PMS2*	P	c.878dup (p.Asn293fs)	29 mut/Mb	High
Patient 25	CRC	MMR‐d	TP	*MSH6*	P	c.1352del (p.Phe451fs)	35 mut/Mb	High
Patient 26	CRC	MMR‐d	TP	*PMS2*	P	c.137G>T (p.Ser46Ile)	20 mut/Mb	High
Patient 27	CRC	MMR‐d	TP	*MSH2*	P	c.942+3A>T	69 mut/Mb	High
Patient 28	CRC	MMR‐d	TP	*PMS2*	P	c.2500_2501delinsG (p.Met834fs)	24 mut/Mb	High
Patient 29	EC	MMR‐d	TP	*MSH6*	P	c.2269_2270del (p.Thr757fs)	20 mut/Mb	Stable
Patient 30	CRC	MMR‐d	TP	*PMS2*	P	c.989‐1_1144+2del	17 mut/Mb	High
Patient 31	EC	MMR‐d and *POLE*	TP	*MSH6*	P	c.3477C>A (p.Tyr1159Ter)	337 mut/Mb	High
Patient 32	CRC	MMR‐d	TP	*MSH2*	P	c.1077‐2A>T	47 mut/Mb	High
Patient 33	CRC	MMR‐d and POLD1	TP	*MSH2*	P	c.968_971dup (p.Gln324fs)	70 mut/Mb	High
Patient 34	CRC	MMR‐d	TP	*PMS2*	P	c.1840A>T (p.Lys614Ter)	33 mut/Mb	High
Patient 35	CRC	MMR‐d	TP	*MLH1*	P	c.588del (p.Lys196fs)	43 mut/Mb	High

*Note:* 35 patients harboring pathogenic or likely pathogenic germline variants in MMR genes identified by clinical germline testing. Tumors were evaluated for somatic mutational signatures, TMB, and MSI. Diag. call reflects concordance between germline pathogenic status and the presence of MMR‐d mutational signature, where TP indicates true positive, and FN indicates false negative. Invitae Sig denotes the variant classification assigned by Invitae at the time of sequencing (P, pathogenic; LP, likely pathogenic). Cancer type is indicated as colorectal cancer (CRC) or endometrial cancer (EC). Genomic HGVS coordinates are reported using GRCh37 reference accessions. Additional clinical and demographic information is provided in Table S2.

### 3.2. Integrating Somatic Data: TMB

The median TMB was 2 mut/Mb (IQR 1–3). Out of the 1110 tumor samples, 148 (13.3%) were MSI‐high and 147 (13.2%) were assigned an MMR‐d mutational signature.

**Figure 3:**
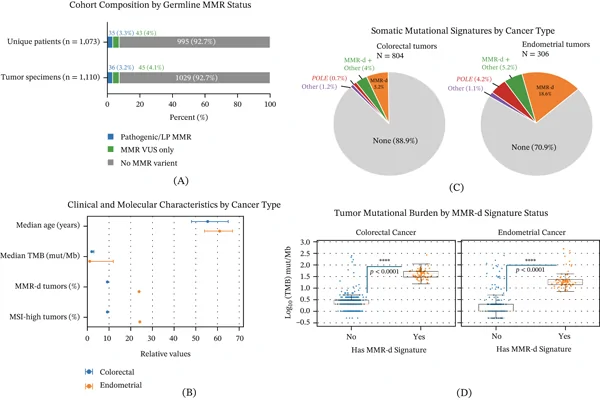
Patient cohort data breakdown. (A) Cohort composition by germline MMR status. Stacked bar plot showing the distribution of germline MMR variant classifications among unique patients (*n* = 1073) and tumor samples (*n* = 1110). Germline variants were classified as pathogenic, VUS or no identified MMR variant based on multigene hereditary cancer panel testing. Bar widths represent percentages within each group. (B) Clinical and molecular characteristics by cancer type. Forest plot summarizing key clinical and molecular features of colorectal cancer (CRC, blue) and endometrial cancer (EC, orange) cohorts. Points denote median values, and horizontal bars represent interquartile ranges where applicable. Variables shown include median age at diagnosis, median TMB, proportion of tumors with an MMR‐deficient (MMR‐d) mutational signature, and proportion of MSI–high tumors. Percentages are calculated per tumor sample. These comparisons highlight differences in age distribution, mutational burden, and prevalence of mismatch repair deficiency between CRC and EC. (C) Distribution of somatic mutational signatures by cancer type. Pie chart showing the percentage of samples stratified by dominant somatic mutational signature category in CRC and EC. Signature categories include tumors with no dominant COSMIC signature, MMR‐d only, MMR‐d in combination with other signatures (e.g., *POLE*, *POLD1*, or HRD), *POLE*‐only hypermutation, and other rare signatures. CRC tumors are predominantly classified as having no dominant signature. In contrast, EC tumors show a substantially higher enrichment for MMR‐d–associated signatures, consistent with known biological differences between these cancer types. (D) Association of MMR‐deficient mutational signatures with TMB in colorectal and EC CRC tumors stratified by the presence or absence of an MMR‐d mutational signature show a marked difference in TMB. Tumors harboring an MMR‐d signature exhibit significantly higher log₁₀‐transformed TMB values compared with tumors lacking an MMR‐d signature. A similar pattern is observed in EC, where tumors with an MMR‐d signature demonstrate significantly increased TMB relative to signature‐negative tumors. In both panels, TMB values were log_10_‐transformed (log_10_[TMB]). Statistical comparisons were performed using the Wilcoxon rank–sum test. Asterisks denote statistical significance ( ^∗∗∗∗^ *p* < 0.0001). Boxplots represent the median and interquartile range, with whiskers extending to 1.5x the interquartile range.

When examined by cancer type, several distinctions emerged. The CRC cohort demonstrated a relatively balanced distribution of sex (372 males and 400 females) and a younger age profile, with a median age of 56 years (IQR 47–65). A total of 233 of the 772 CRC patients (30.2%) were classified as early‐onset. CRC tumors had a median TMB of 2 mut/Mb (IQR 2–3), consistent with the expected mutational landscape of CRC. MMR‐d signatures were observed in 74 of 804 CRC samples (9.2%) (Figure 3B).

In contrast, the EC cohort exhibited an older age distribution, with a median age of 61 years (IQR 54–67). Only 46 of 301 EC patients (15.3%) were classified as early‐onset. Median TMB was 1 mut/Mb, but the interquartile range was considerably wider (1–12 mut/Mb) compared with CRC (2–3 mut/Mb). This broad distribution reflects the molecular heterogeneity of EC, including the presence of hypermutated tumors driven by MMR‐d and *POLE* disruption. Consistent with this, MMR‐d signatures were substantially more common in EC than CRC, identified in 73 of 306 samples (23.9%). MSI‐high status showed a similar enrichment in EC (24.2%) compared with CRC (9.2%), further supporting the higher underlying burden of MMR deficiency in endometrial tumors (Figure 3B).

### 3.3. Distribution of Somatic Mutational Signatures

COSMIC SBS signature analysis revealed that most tumors in the combined cohort did not exhibit a dominant mutational process, with 932 of 1110 samples (83.9%) classified as having no identifiable signature. Among tumors with a detectable signature, MMR–associated patterns were the most common. A total of 99 tumors (8.9%) exhibited a pure MMR‐d signature, and an additional 48 tumors (4.3%) showed MMR‐d in combination with other processes. These mixed signatures were primarily MMR‐d plus *POLD1*‐associated patterns (3.1%) or MMR‐d plus *POLE*‐associated patterns (1%), although rare combinations were also observed (Table 3). Other non‐MMR signatures, including isolated *POLE* hypermutation (1.7%), homologous recombination deficiency (0.3%), *APOBEC* activity (0.1%), and *MUTYH*‐associated mutagenesis (0.1%), were uncommon. (Figure 3C).

**Table 3:** Clinicopathologic and somatic features of tumors from patients harboring germline MMR VUS.

ID	Signature	Cancer	Gene	Invitae	HGVS	TMB score	MSI	Site
143	None	EC	*MSH2*	VUS	c.1567T>G (p.Phe523Val)	1 mut/Mb	Stable	Peritoneum
144	None	CRC	*PMS2*	VUS	c.1522A>G (p.Ile508Val)	1 mut/Mb	Stable	Liver
145	None	EC	*MSH6*	VUS	c.2960C>G (p.Thr987Ser)	1 mut/Mb	Stable	Uterus
146	None	CRC	*PMS2*	VUS	c.886C>T (p.Pro296Ser)	1 mut/Mb	Stable	Rectum
147	None	CRC	*PMS2*	VUS	c.2504G>A (p.Gly835Glu)	1 mut/Mb	Stable	Omentum
147	None	CRC	*PMS2*	VUS	c.2504G>A (p.Gly835Glu)	2 mut/Mb	Stable	Colon
148	None	EC	*MSH6*	VUS	c.490C>G (p.His164Asp)	1 mut/Mb	Stable	Uterus
149	None	CRC	*MSH6*	VUS	c.261‐3C>T	1 mut/Mb	Stable	Cecum
150	None	CRC	*MSH6*	VUS	c.1825C>A (p.Leu609Ile)	1 mut/Mb	Stable	Colon
151	None	CRC	*MLH1*	VUS	c.2242G>C (p.Asp748His)	1 mut/Mb	Stable	Colon
152	None	EC	*MLH1*	VUS	c.1732G>A (p.Glu578Lys)	1 mut/Mb	Stable	Endometrium
152	None	EC	*MLH1*	VUS	c.1935C>G (p.Asn645Lys)	1 mut/Mb	Stable	Endometrium
153	None	EC	*MSH6*	VUS	c.113C>T (p.Pro38Leu)	1 mut/Mb	Stable	Uterus
154	MMR‐d/*POLE*	EC	*MSH2*	VUS	c.2768T>A (p.Val923Glu)	108 mut/Mb	High	Endometrium
154	MMR‐d/*POLE*	EC	*MSH6*	VUS	c.3563G>A (p.Ser1188Asn)	108 mut/Mb	High	Endometrium
155	None	CRC	*PMS2*	VUS	c.2243_2246del (p.Lys748fs	2 mut/Mb	Stable	Skin
156	None	CRC	*MSH2*	VUS	c.1087G>T (p.Val363Leu)	2 mut/Mb	Stable	Rectum
157	None	CRC	*MSH6*	VUS	c.359T>C (p.Ile120Thr)	2 mut/Mb	Stable	Omentum
157	None	CRC	*MSH6*	VUS	c.359T>C (p.Ile120Thr)	4 mut/Mb	Stable	Soft Tissue
158	None	CRC	*MSH6*	VUS	c.3163G>A (p.Ala1055Thr)	2 mut/Mb	Stable	Colon
159	None	CRC	*MSH6*	VUS	c.2061T>G (p.Cys687Trp)	2 mut/Mb	Stable	Colon
160	None	CRC	*PMS2*	VUS	c.883C>T (p.Arg295Trp)	2 mut/Mb	Stable	Colon
161	None	CRC	*PMS2*	VUS	c.961GTT[1] (p.Val322del)	2 mut/Mb	Stable	Colon
162	None	CRC	*MSH2*	VUS	c.2354A>G (p.His785Arg)	2 mut/Mb	Stable	Lung
163	None	CRC	*MSH6*	VUS	c.1190A>G (p.Tyr397Cys)	2 mut/Mb	Stable	Retroperitoneal
164	None	CRC	*MSH6*	VUS	c.869T>C (p.Leu290Pro)	2 mut/Mb	Stable	Colon
165	None	CRC	*MSH2*	VUS	c.756G>C (p.Gln252His)	2 mut/Mb	Stable	Small Intestine
166	None	CRC	*MSH6*	VUS	c.4004A>C (p.Glu1335Ala)	2 mut/Mb	Stable	Colon
167	None	CRC	*PMS2*	VUS	c.1936A>G (p.Arg646Gly)	2 mut/Mb	Stable	Soft Tissue
168	None	EC	*MSH6*	VUS	c.4068G>C (p.Leu1356Phe)	2 mut/Mb	Stable	Uterus
169	None	CRC	*PMS2*	VUS	c.961G>A (p.Val321Ile)	2 mut/Mb	Stable	Colon
170	None	EC	*MLH1*	VUS	c.237G>T (p.Arg79Ser)	2 mut/Mb	Stable	Uterus
171	None	CRC	*MSH6*	VUS	c.2300C>G (p.Thr767Ser)	2 mut/Mb	Stable	Rectum
172	None	CRC	*MSH6*	VUS	c.2028A>T (p.Lys676Asn)	2 mut/Mb	Stable	Colon
173	MMR‐d/*POLD1*	EC	*MSH6*	VUS	c.877C>T (p.Pro293Ser)	21 mut/Mb	High	Uterus
174	MMR‐d	EC	*PMS2*	VUS	c.1463C>T (p.Ala488Val)	25 mut/Mb	High	Uterus
175	None	CRC	*PMS2*	VUS	c.2559C>G (p.Ile853Met)	3 mut/Mb	Stable	Colon
176	None	CRC	*MSH6*	VUS	c.821G>A (p.Ser274Asn)	3 mut/Mb	Stable	Colon
177	None	CRC	*MSH2*	VUS	c.1582A>C (p.Lys528Gln)	3 mut/Mb	Stable	Colon
178	MMR‐d	CRC	*MLH1*	VUS	c.185A>C (p.Gln62Pro)	30 mut/Mb	High	Colon
179	MMR‐d/*POLD1*	CRC	*MSH6*	VUS	c.4000C>T (p.Arg1334Trp)	30 mut/Mb	High	Colon
180	None	CRC	*PMS2*	VUS	c.1399G>A (p.Val467Ile)	4 mut/Mb	Stable	Rectum
181	None	CRC	*PMS2*	VUS	c.2554C>T (p.His852Tyr)	4 mut/Mb	Stable	Brain
182	None	CRC	*MLH1*	VUS	c.1832T>C (p.Ile611Thr)	4 mut/Mb	Stable	Liver
183	None	CRC	*PMS2*	VUS	c.794A>G (p.Asn265Ser)	4 mut/Mb	Stable	Ovary
184	None	CRC	*PMS2*	VUS	c.1733G>A (p.Arg578His)	51 mut/Mb	High	Liver
185	MMR‐d	CRC	*MLH1*	VUS	c.1877T>C (p.Phe626Ser)	57 mut/Mb	High	Colon

*Note:* tumors from patients with germline VUS in MMR genes identified by clinical germline testing. Tumors were evaluated for somatic mutational signatures, TMB, and MSI. The Signature column indicates the presence or absence of an MMR‐d mutational signature, including cases with combined MMR‐d and *POLE*/*POLD1*‐associated signatures where applicable. Invitae Sig denotes the germline variant classification assigned by Invitae at the time of sequencing. Cancer type is reported as colorectal cancer (CRC) or endometrial cancer (EC). HGVS nomenclature includes both cDNA‐level (c.) and protein‐level (p.) annotations when applicable and follows GRCh38 reference transcripts. Some patients contribute more than one entry due to the sequencing of multiple independent tumors. Additional variant interpretation details and Bayesian scoring are provided in subsequent tables and the supporting data.

Stratification by cancer type showed distinct differences in mutational processes. Colorectal tumors were predominantly classified as “None” (88.9%), with 42 tumors (5.2%) exhibiting a pure MMR‐d signature and another 4% showing mixed signatures that included MMR‐d with *POLE, POLD1,* or HRD features. In contrast, ECs displayed a substantially higher prevalence of MMR–associated signatures. Only 70.9% of EC tumors lacked a dominant signature, whereas 18.6% showed a pure MMR‐d signature and 5.2% demonstrated mixed MMR‐d plus *POLE* or *POLD1* signatures. Isolated *POLE* hypermutation was also more frequent in EC (4.2%) than in CRC (Figure 3C).

Together, these findings indicate that MMR–deficient mutational processes, whether occurring alone or in combination with other hypermutator pathways, represent the most common identifiable signatures in this cohort. This pattern was especially prominent in ECs, which contributed disproportionately to the overall burden of MMR‐d and hypermutated tumors.

### 3.4. Comparison of MMR‐d Signature by Cancer Type

In addition to classifying mutational processes, we compared TMB between tumors with and without an MMR‐d signature within each cancer type. In both colorectal and EC cohorts, MMR‐d tumors exhibited sharply elevated TMB relative to MMR‐proficient tumors, consistent with the biological consequences of MMR failure. However, the magnitude of this elevation differed between cancer types. In CRC, MMR‐d tumors demonstrated exceptionally high TMB levels, with a log‐transformed distribution noticeably higher than that of MMR‐d endometrial tumors (Figure 3D). By contrast, ECs showed a broader overall TMB distribution, with an interquartile range spanning 1–12 mut/Mb compared with 2–3 mut/Mb in CRCs. This wider range in EC reflects a larger presence of hypermutated subsets, like *POLE*‐mutated ultramutated tumors that elevate the overall variability in TMB independently of MMR status. As a result, although colorectal MMR‐d tumors tend to reach higher TMB levels on a per‐tumor basis, ECs remain more heterogeneous overall, with a larger fraction of hypermutated tumors arising from multiple underlying biological mechanisms. Together, these findings illustrate that although MMR‐d universally drives increased TMB, the broader mutational landscape of EC leads to greater overall variability in hypermutation patterns across the EC cohort.

### 3.5. Association Between Somatic MMR‐d Signature and Germline Pathogenic MMR Variants

**Figure 4:**
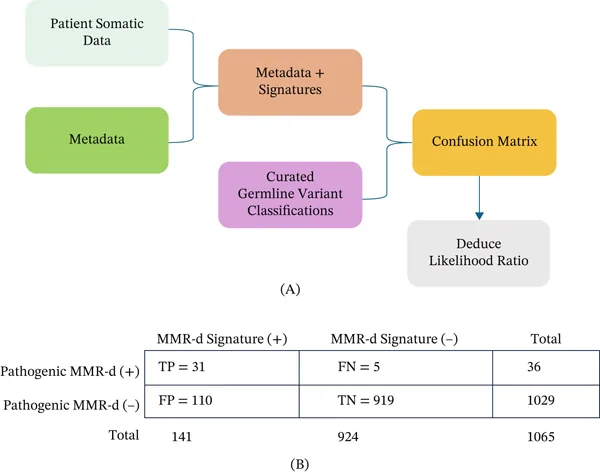
A) Somatic mutational signature analysis workflow for likelihood ratio (LR) derivation. Workflow for deriving and interpreting LRs from somatic mutational signature analysis. Somatic variant calls were used to calculate COSMIC single‐base substitution (SBS) signatures and merged with clinical metadata and Invitae germline variant data. Each patient was annotated for the presence of a pathogenic MMR germline variant (*MLH1*, *PMS2*, *MSH2*, and/or *MSH6*) and an MMR‐deficient (MMR‐d) signature. A contingency table was constructed to classify true‐positive, false‐positive, true‐negative, and false‐negative observations, from which LRs were computed to quantify how strongly MMR‐d signature presence predicts an underlying germline pathogenic variant. The conceptual interpretation of LR magnitude as “Supporting,” “Moderate,” or “Strong” evidence strength follows the Bayesian ACMG/AMP calibration proposed by Tavtigian et al., [23, 24]. However, the LRs themselves were derived empirically from this dataset. (B) Association between MMR‐d mutational signature and pathogenic germline MMR variants. 2 × 2 contingency structure used to evaluate the association between the presence of a mismatch repair–deficient (MMR‐d) mutational signature in tumors and confirmed pathogenic germline variants in MMR genes.

To quantify the strength of the association between the presence of an MMR‐d mutational signature and an underlying germline pathogenic variant in *MLH1*, *PMS2*, *MSH2*, or *MSH6*, we performed an association analysis using the integrated somatic‐germline dataset (Figure 4A).

Across 1065 evaluable samples, 31 tumors with a germline pathogenic MMR variant also displayed an MMR‐d signature, whereas 5 tumors with a germline pathogenic variant did not. Conversely, 919 tumors lacked both a germline pathogenic variant and an MMR‐d signature, and 110 tumors without a pathogenic variant demonstrated an MMR‐d signature, reflecting the biological complexity of alternative factors that lead to an MMR‐d signature (i.e., somatic mutations and somatic *MLH1* hypermethylation). A total of 36 tumors from patients with at least one germline MMR pathogenic variant were matched to 35 unique patients (one patient had two tumor samples sequenced). Of the 35 patients, 12/35 had a *PMS2* mutation, 7/35 had an *MSH6* mutation, 7/35 had an *MSH2* mutation, and 9/35 had an *MLH1* mutation (Table 2; Figure S2B). Of the 35 patients with germline pathogenic MMR variants, 26 had CRC, contributing 27 CRC tumor specimens, and 9 had EC, contributing 9 EC tumor specimens. TMB among these patients was markedly elevated in many cases, with a median TMB of 31 mut/Mb (IQR: 21–47 mut/Mb). Notably, several tumors exhibited ultrahypermutation (> 100 mut/Mb), with at least one of those cases also having a signature for *POLE* deficiency, which is known for very highly mutated tumors.

From this confusion matrix distribution, the sensitivity of the MMR‐d signature for identifying tumors in individuals with a germline pathogenic MMR variant was 86.1% (95% CI, 71%–95%), and the specificity was 89.3% (95% CI, 87%–91%). This yielded an LR+ of 8.06 (95% CI, 6.46–10.04), corresponding to a log10 LR+ of 0.90 (95% CI, 0.81–1.00). The LR−, calculated as (1 − sensitivity)/specificity, was 0.156 (95% CI, 0.069–0.351), corresponding to a log10 LR− of −0.81 (95% CI, −1.16 to −0.45) (Figure S3). According to the Bayesian ACMG/AMP calibration framework proposed by Tavtigian and colleagues, an LR+ of this magnitude constitutes moderate‐to‐strong evidence supporting pathogenicity, whereas the absence of an MMR‐d signature provides moderate‐to‐strong benign evidence.

Importantly, this estimate reflects the biological reality that although many tumors with MMR‐d signatures arise from somatic mechanisms (e.g., *MLH1* promoter hypermethylation or double somatic MMR gene inactivation), the presence of an MMR‐d mutational signature nevertheless increases the probability of an underlying germline pathogenic MMR variant by approximately eightfold. Thus, although the absence of an MMR‐d signature strongly argues against pathogenicity, its presence provides substantial, though not determinative, evidence when incorporated alongside orthogonal molecular data within a Bayesian reclassification framework.

As an example of the advantage of this Bayesian approach, we noted that of the 31 TP cases, Patients 7 and 29 presented with germline pathogenic *MSH6* variants, elevated TMB, but were microsatellite stable (MSS) (Table 2). IHC of the MMR proteins for these patients confirmed loss of *MSH6* protein in both patients, supporting the pathogenicity of the *MSH6* variants. Unlike MSI testing, which interrogates a limited set of loci, mutational signatures capture the global exonic consequence of the repair deficiency, thus capturing cases that may have been missed by microsatellite testing.

### 3.6. Discordant Cases With Germline Pathogenic MMR Variants and MMR‐d Signature

To understand the factors contributing most to the sensitivity and specificity, we closely examined the 110 tumors that showed MMR‐d signature in the absence of pathogenic germline MMR gene variant (FPs) (Table S1), and 5 tumors with pathogenic germline MMR variant and no MMR‐d signature (FNs) (Table 2 and Table S2). Of the 110 false‐positive tumor specimens, 107 unique patients were represented, 63 specimens were from EC, and 47 were from CRC. The median TMB was 23 mut/Mb (IQR: 15.5–43 mut/Mb). Of the 110 samples, 54 tumors had at least one somatic mutation in an MMR gene, with 18 of those having either multiple somatic hits in the same MMR gene or in different genes.

Of the five false‐negative tumors, two had low TMB and MSS profiles, suggesting that they represented sporadic MMR‐proficient tumors arising in individuals with Lynch syndrome [47]. A third tumor had an intermediate TMB of 11 mut/Mb but lacked an MMR‐d signature in the COSMIC v3.6 analysis. This specimen had shown low‐level SBS15 attribution in the prior v3.4 analysis, but no MMR‐d‐associated signature met the v3.6 calling criteria. The remaining two discordant tumors had high TMB, MSI‐high status, and immunohistochemical loss of the protein corresponding to the respective germline pathogenic variant. These cases suggest that tumor evolution, sampling differences, signature assignment thresholds, or additional somatic events can produce MMR‐d phenotypes that do not yield a clear COSMIC MMR‐d signature under the applied calling criteria.

This LR framework formed the basis for downstream evaluation of the 45 distinct germline MMR VUS identified in 43 patients, allowing application of the empirically derived LR to variant‐specific prior probabilities. This enables quantitative assessment of whether the presence of an MMR‐d signature meaningfully increases the posterior probability of pathogenicity for individual VUSs, and whether certain VUS‐harboring tumors behave analogously to known pathogenic cases.

### 3.7. Integrative Bayesian Reclassification of Germline VUS in MMR Genes

We then applied this Bayesian framework to 45 distinct germline MMR VUS identified in 43 patients and represented across 45 tumor specimens (Table 4). For each case, we combined tumor mutational signature status, somatic mutation data (such as whether the patient had a somatic second hit of the same gene as the germline VUS), allelic expression, methylation, IHC, and curated variant‐level evidence (including population frequency, in silico predictions, and functional studies) to calculate variant‐specific posterior probabilities (Table 1). This method allowed systematic reclassification of VUS as likely pathogenic, likely benign, or remaining as VUS, though with greater clarity about the favored direction.

**Table 4:** Posterior probability changes for germline MMR VUS following integration of somatic evidence.

Gene	Variant (HGVS)	Initial	Prior	Evidence Added (abbreviated)	Posterior	Final
*MLH1*	p.Asp748His	VUS	10%	PM2_supp., No MMR‐d Sig.	3.51%	Likely benign
*MLH1*	p.Glu578Lys	VUS	10%	PM2_supp., PS4_supp., No MMR‐d Sig.	7.10%	Likely benign
*MLH1*	p.Asn645Lys	VUS	10%	PM2_mod., No MMR‐d Sig.	6.94%	Likely benign
*MLH1*	p.Arg79Ser	VUS	10%	PP3_supp., No MMR‐d Sig.	3.51%	Likely benign
*MLH1*	p.Ile611Thr	VUS	10%	No MMR‐d Sig.	1.70%	Likely benign
*MSH2*	p.Val363Leu	VUS ^∗^	10%	PP3_supp., No MMR‐d Sig.	3.51%	Likely benign
*MSH2*	p.His785Arg	VUS ^∗^	10%	PM2_supp., PP3_supp., No MMR‐d Sig.	7.10%	Likely benign
*MSH2*	p.Gln252His	VUS ^∗^	10%	PM2_supp., No MMR‐d Sig.	3.51%	Likely benign
*MSH2*	p.Lys528Gln	VUS ^∗^	10%	PM2_supp., PP3_supp., No MMR‐d Sig.	7.10%	Likely benign
*MSH6*	p.Thr987Ser	VUS	10%	PM2_mod., No MMR‐d Sig.	6.94%	Likely benign
*MSH6*	p.His164Asp	VUS	10%	PM2_mod., No MMR‐d Sig.	6.94%	Likely benign
*MSH6*	c.261‐3C > T	VUS	10%	PM2_mod., No MMR‐d Sig.	6.94%	Likely benign
*MSH6*	p.Leu609Ile	VUS ^∗^	10%	PM2_supp., No MMR‐d Sig.	3.51%	Likely benign
*MSH6*	p.Pro38Leu	VUS	10%	PM2_mod., No MMR‐d Sig.	6.94%	Likely benign
*MSH6*	p.Ile120Thr	VUS ^∗^	10%	PM2_supp., No MMR‐d Sig.	3.51%	Likely benign
*MSH6*	p.Ala1055Thr	VUS ^∗^	10%	PM2_supp. PS4_supp., No MMR‐d Sig.	7.10%	Likely benign
*MSH6*	p.Cys687Trp	VUS	10%	PM2_mod., No MMR‐d Sig.	6.94%	Likely benign
*MSH6*	p.Tyr397Cys	VUS ^∗^	10%	PM2_supp., PP3_supp., No MMR‐d Sig.	7.10%	Likely benign
*MSH6*	p.Leu290Pro	VUS ^∗^	10%	PM2_supp., No MMR‐d Sig.	3.51%	Likely benign
*MSH6*	p.Glu1335Ala	VUS ^∗^	10%	No MMR‐d Sig.	1.70%	Likely benign
*MSH6*	p.Leu1356Phe	VUS ^∗^	10%	PM2_supp., No MMR‐d Sig.	3.51%	Likely benign
*MSH6*	p.Thr767Ser	VUS ^∗^	10%	PM2_supp., PP3_supp., No MMR‐d Sig.	7.10%	Likely benign
*MSH6*	p.Lys676Asn	VUS	10%	PM2_mod., No MMR‐d Sig.	6.94%	Likely benign
*MSH6*	p.Ser274Asn	VUS ^∗^	10%	PP3_supp., No MMR‐d Sig.	3.51%	Likely benign
*PMS2*	p.Ile508Val	VUS	10%	PM2_mod., No MMR‐d Sig.	6.94%	Likely benign
*PMS2*	p.Pro296Ser	VUS	10%	PM2_supp., No MMR‐d Sig.	3.51%	Likely benign
*PMS2*	p.Gly835Glu	VUS	10%	PP3_supp., No MMR‐d Sig.	3.51%	Likely benign
*PMS2*	p.Arg295Trp	VUS	10%	PS4_supp., PP3_supp., No MMR‐d Sig.	7.10%	Likely benign
*PMS2*	p.Val322del	VUS	10%	PM2_mod., No MMR‐d Sig.	6.94%	Likely benign
*PMS2*	p.Val321Ile	VUS	10%	PS4_supp., No MMR‐d Sig.	3.51%	Likely benign
*PMS2*	p.Ile853Met	VUS ^∗^	10%	No MMR‐d Sig.	1.70%	Likely benign
*PMS2*	p.Val467Ile	VUS ^∗^	10%	PS4_supp., No MMR‐d Sig.	3.51%	Likely benign
*PMS2*	p.His852Tyr	VUS	10%	No MMR‐d Sig.	1.70%	Likely benign
*PMS2*	p.Asn265Ser	VUS	10%	No MMR‐d Sig.	1.70%	Likely benign
*PMS2*	p.Arg578His	VUS ^∗^	10%	No MMR‐d sig	1.70%	Likely benign
*MLH1*	p.Gln62Pro	VUS	10%	PM2_mod., MMR‐d sig., 2^nd^ Hit, Mutant ASE, IHC_mod.	99.33%	Pathogenic
*MLH1*	p.Phe626Ser	VUS	10%	PM2_mod., PP3_supp., MMR‐d sig., 2nd Hit, IHC mod.	99.30%	Pathogenic
*MSH6*	p.Ser1188Asn	VUS	10%	PM2_mod., PS3_supp., PS4_supp., PP3_supp., 2^nd^ Hit	95.00%	Likely pathogenic
*MSH2*	p.Val923Glu	VUS	10%	PM2_supp., PP3_supp., PS4_supp.	50.71%	VUS (unchanged)
*MSH2*	p.Phe523Val	VUS	10%	PM2_mod., No MMR‐d Sig., PP3_supp.	13.53%	VUS (unchanged)
*MSH6*	p.Pro293Ser	VUS ^∗^	10%	PM2_supp., MMR‐d sig., BP5_strong	15.70%	VUS (unchanged)
*MSH6*	p.Arg1334Trp	VUS∗	10%	PM2_supp., MMR‐d sig., 2^nd^ Hit, BP5_strong	44.50%	VUS (unchanged)
*PMS2*	p.Ala488Val	VUS	10%	PM2_supp., MMR‐d sig., BP5_mod.	28.16%	VUS (unchanged)
*PMS2*	p.Lys748fs	VUS ^∗∗^	10%	PM2_mod., PP3_supp., No MMR‐d sig.	13.53%	VUS (unchanged)
*PMS2*	p.Arg646Gly	VUS	10%	PM2_mod., PP3_supp., No MMR‐d sig	13.53%	VUS (unchanged)

*Note:* Bayesian posterior probability updates for 45 germline MMR gene variants initially classified as VUS. Posterior probabilities were calculated by sequential integration of somatic and supporting evidence, including population frequency (PM2), case enrichment (PS4), benign evidence (BS2), presence or absence of an MMR‐d mutational signature, IHC loss patterns, and allele‐specific expression. The Final classification reflects the posterior interpretation following incorporation of all available evidence. Evidence weights and corresponding log_10_ likelihood ratios are defined in Table 1. Variants marked with an asterisk ( ^∗^) were subsequently reclassified by Invitae as Likely Benign or Benign, although reported as VUS at the time of sequencing. Variants marked with a double asterisk ( ^∗∗^) have Likely Pathogenic submissions in ClinVar, whereas Invitae retains a VUS classification.

As established in the preceding section, the presence of an MMR‐d mutational signature increased the likelihood of an underlying pathogenic germline MMR variant by approximately eightfold (LR + ≈8; log10 LR ≈ 0.9). This magnitude corresponds to moderate‐to‐strong pathogenic evidence under the Tavtigian Bayesian calibration and represents a biologically grounded measure of tumor‐level MMR failure. Importantly, although the MMR‐d signature alone was insufficient to establish pathogenicity, given the substantial proportion of MMR‐d tumors driven by somatic mechanisms, it consistently shifted posterior probabilities upward when applied to germline VUS with otherwise equivocal evidence. Conversely, the absence of an MMR‐d signature provided moderate‐to‐strong benign evidence (LR − ≈0.156), rapidly decreasing posterior probabilities, and enabled confident downgrading of multiple VUS.

Of the 45 distinct VUS evaluated, 38 were represented among 39 MMR‐d‐signature‐negative tumor specimens, whereas 7 were represented among 6 MMR‐d‐signature‐positive tumors. The MMR‐d‐negative tumors had a median TMB of 2 mut/Mb (IQR, 1–2 mut/Mb). One MMR‐d‐negative tumor was MSI‐high with elevated TMB but retained expression of the MMR proteins by IHC; the remaining MMR‐d‐negative tumors were MSS. The six MMR‐d‐positive tumors had a median TMB of 30 mut/Mb (IQR, 26.25–50.25 mut/Mb) and were all MSI‐high (Table 3 and Table S3). Using our Bayesian framework, we were able to show evidence of posterior probability of under 10% for 35 of the 45 variants that were deemed a VUS by Invitae at the time of the sequencing of the patient (Table 4). Interestingly, Invitae has since reclassified 18 of those variants as “likely benign” or “benign,” of which 16 of them are variants that this analysis downgraded, underscoring the validity of this integrative approach (Table 4). Interestingly, one of the variants called a VUS by Invitae, *PMS2* p.Lys748fs, was deemed “Pathogenic” by the International Society for Gastrointestinal Hereditary Tumours. Our final calculation revealed that, given the tumor′s lack of MMR‐d signature (LR − ≈0.156), along with in silico data (PP3_supporting) and population data (PM2_moderate), the final posterior probability remained above the 10% to retain the “VUS” status. Important to note that the tumor sample for this CRC patient came from the skin. (Table 4). Furthermore, our analysis revealed that three of the called VUSs Invitae have evidence for a final posterior probability above the 90% threshold to be likely pathogenic. In fact, two of those variants have a posterior probability of over 99%, which pushes the status to pathogenic.

### 3.8. Examination of *MLH1* c.185A>C (p.Gln62Pro) Upgrade

The first upgraded variant was *MLH1* c.185A>C (p.Gln62Pro), identified in a 68‐year‐old man with CRC (TMB 30 mut/Mb, MSI‐high). At the time of clinical testing, Invitae classified this missense change as a VUS; it is absent from gnomAD, with no published segregation or functional data, corresponding to PM2_moderate plus no strong counterevidence. Using Tavtigian′s prior of 10% [23] for an MMR VUS, PM2_moderate alone increased the posterior probability to ~0.32.

In our cohort, the tumor showed a clear MMR‐d mutational signature and a high TMB, with a somatic second hit in *MLH1* (frameshift/LOF, AF 11%), consistent with biallelic inactivation. Mutant allele–specific expression analysis demonstrated enrichment of the mutant allele in tumor RNA relative to tumor DNA, supporting preferential expression from the mutant allele (mutant ASE). IHC showed concordant loss of *MLH1*/*PMS2* with intact *MSH2*/*MSH6* and no evidence of *MLH1* promoter hypermethylation, providing moderate functional evidence that loss of *MLH1* is the primary driver lesion.

Within the Bayesian scheme, these additional data contributed the following: LR ≈ 8 for MMR‐d signature (supporting pathogenic), LR ≈ 4.3 for the *MLH1* second hit (PS3_moderate–style mechanistic evidence), LR ≈ 2.1 for mutant ASE (supporting functional impact), and LR ≈ 4.3 for moderate IHC evidence (PS3_mod). Multiplying these by the prior updated by PM2_moderate yielded a final posterior probability of 99.3%, crossing the ACMG/AMP threshold for “pathogenic”. In the trajectory plot (Figure 2), Invitae evidence alone brings the variant only into the ~0.32 range, whereas the addition of MMR‐d signature, second hit, ASE, and IHC moves it decisively into the “pathogenic” range.

### 3.9. Examination of *MLH1* p.Phe626Ser (NC_000003.11:g.37089155T>C) upgrade

The second upgraded variant was *MLH1* c.1877T>C (p.Phe626Ser) in a 74‐year‐old man with CRC (TMB 57 mut/Mb, MSI‐high). This variant is also absent from gnomAD and was originally classified by Invitae as a VUS. Advanced in silico modeling predicted a damaging effect on *MLH1* function, supporting PP3_supporting, in addition to PM2_moderate, which together increased the posterior probability to ~0.5 under a 10% prior.

Tumor profiling again demonstrated a robust MMR‐d mutational signature with high TMB and a somatic loss‐of‐function second hit in *MLH1* (AF 14%). IHC revealed loss of *MLH1/PMS2* with intact *MSH2/MSH6* and no detectable *MLH1* promoter hypermethylation, matching the germline gene and arguing against an alternative epigenetic mechanism. The combination of MMR‐d signature, biallelic *MLH1* inactivation, and *MLH1*‐specific IHC loss provided convergent evidence that the germline variant is functionally deleterious.

This variant was absent from gnomAD and was predicted to have a damaging effect on MLH1 function, supporting PM2_moderate and PP3_supporting evidence, respectively. Starting from a 10% prior, PM2_moderate increased the posterior probability to 32.3%, and addition of PP3_supporting increased it to 50.1%. Incorporation of the MMR‐d signature increased the posterior probability to 88.9%, whereas addition of the somatic loss‐of‐function second hit increased it to 97.2%. Concordant *MLH1*/*PMS2* loss by IHC, in the absence of detectable MLH1 promoter hypermethylation, resulted in a final posterior probability of approximately 99.3%, supporting pathogenic classification.

### 3.10. Examination of *MSH6* p.Ser1188Asn (NC_000002.11:g.48032763G>A) upgrade

The third upgraded variant, *MSH6* c.3563G>A (p.Ser1188Asn), was identified in a 65‐year‐old woman with endometrial carcinoma characterized by high TMB and MSI. The variant is absent from gnomAD (PM2_moderate) and has been reported in multiple CRC cases [48, 49]. In silico modeling predicts a deleterious effect (PP3_supporting), and independent functional studies demonstrate MMR‐d in vitro (PS3_supporting) [49]. These germline and functional data alone increase the posterior probability to approximately 0.8 under a 10% prior.

Tumor sequencing revealed a clonal somatic frameshift mutation in *MSH6* at an AF of 55%, consistent with biallelic inactivation of *MSH6*. IHC demonstrated concurrent loss of *MSH2* and *MSH6* with intact *MLH1* and *PMS2*. Although this staining pattern is classically associated with *MSH2* pathogenicity, prior experimental and clinical studies have shown that disruptive variants affecting the C‐terminal interaction domain of *MSH6* can destabilize the MutS*α* complex and result in secondary loss of *MSH2* protein, representing a known but uncommon exception to the typical immunohistochemical pattern [38, 49]. In this context, the immunohistochemical findings were interpreted as supportive of MMR‐d but not gene‐specific.

This tumor also harbored a germline *MSH2* VUS, *MSH2* c.2768T>A (p.Val923Glu). Because both variants affect components of the same heterodimeric complex, tumor‐level features, including IHC and the MMR‐d signature, could not be conclusively attributed to one variant over the other. Accordingly, although the MMR‐d signature was observed, it was not incorporated as variant‐specific evidence in the Bayesian calculation. When combining PM2 at moderate strength, PS3 and PP3 at supporting strength, PS4 at supporting strength, and the somatic second hit as moderate mechanistic evidence, the posterior probability for *MSH6* p.Ser1188Asn reached 95%, consistent with a likely pathogenic classification.

In contrast, for the co‐occurring *MSH2* p.Val923Glu variant, only germline‐level evidence was incorporated into the Bayesian framework. This included PM2_supporting based on a gnomAD allele frequency of 0.0009%, PS4_supporting based on reports in individuals with Lynch syndrome, and PP3_supporting based on in silico predictions of deleterious effect. These data yielded a posterior probability of 50.7%, and the variant was therefore retained as a VUS.

### 3.11. Additional VUS Changes

Of the remaining four VUSs, three (*MSH6* p.Pro293Ser: NC_000002.11:g.48025999C>T; *MSH6* p.Arg1334Trp: NC_000002.11:g.48033789C>T; and *PMS2* p.Ala488Val: NC_000007.13:g.6026933G>A) were in patients with somatic *MLH1* promoter hypermethylation and corresponding IHC data, which showed *MLH1/PMS2* degradation. For the two patients who had a germline VUS in *MSH6*, the lack of degradation of *MSH2/MSH6* but loss of *MLH1/PMS2* suggests BP5_strong (LR = 0.1) provides strong evidence for the variants being benign (Table 3, Table S3). As *PMS2* complexes with *MLH1*, it is not conclusive to suggest that *MLH1* hypermethylation is driving protein degradation, so this drops this variant to BP5_moderate (LR = 0.21) (Table 3, Table S3).

Together, these cases demonstrate that although an MMR‐d mutational signature meaningfully increases the probability that a germline MMR variant is pathogenic, gene‐concordant somatic evidence and IHC patterns are critical discriminators. In the absence of biallelic inactivation, mutant allele–specific expression, or IHC loss attributable to the same gene as the germline VUS, MMR‐d signatures alone were insufficient to justify variant upgrade and instead appropriately supported either VUS retention or benign reclassification.

### 3.12. Sensitivity Analyses of Panel‐Restricted Mutational Signatures and TMB

To assess whether the association between MMR‐d mutational signatures and pathogenic germline MMR variants could be recovered from targeted sequencing data, we repeated the analysis after restricting somatic variants to the FoundationOne CDx 324‐gene coding footprint. Panel‐restricted variants were fitted using a FoundationOne CDx trinucleotide‐renormalized COSMIC v3.6 reference catalogue and were matched to cohort specimens using tumor‐specific identifiers. A total of 11 specimens with no panel‐overlapping SBS variants were retained as panel‐MMR‐d negative; none of these specimens originated from patients with pathogenic germline MMR variants.

Direct transfer of the exome‐derived activity threshold to panel‐restricted data resulted in a marked loss of sensitivity. For example, applying a threshold of at least 200 cumulative MMR‐d‐associated mutations identified only 1 of 36 pathogenic germline MMR carriers. We therefore recalibrated panel MMR‐d status using the summed attributed activity across SBS6, SBS14, SBS15, SBS20, SBS21, SBS26, and SBS44.

At the maximum Youden′s J operating point **[** 37], defined as a summed MMR‐d activity threshold of at least 5, panel‐restricted analysis yielded a sensitivity of 58.3%, specificity of 91.4%, LR+ of 6.74, and LR− of 0.456. At a more stringent threshold of at least 8, selected to maximize LR+ while retaining sensitivity above 50%, sensitivity was 52.8% and specificity was 94.5%, yielding LR+ of 9.53 and LR− of 0.500. Although the higher threshold produced an LR+ comparable to or slightly greater than the whole‐exome estimate of 8, this reflected increased specificity at the cost of substantially reduced sensitivity. Thus, panel‐restricted signatures could provide moderate positive evidence when an MMR‐d signal was detected, but panel‐negative results provided little evidence against pathogenicity. The diagnostic performance of the whole‐exome MMR‐d signature classifier, including sensitivity, specificity, LR+, LR−, and log10‐transformed LRs with 95% CIs, is summarized in Figure S3.

We also evaluated TMB as an alternative tumor‐level correlate of pathogenic germline MMR status. Tumors classified as intermediate or high TMB, corresponding to at least 10 mutations per megabase, were considered TMB‐positive. TMB‐positive status identified 34 of 36 pathogenic germline MMR carrier tumors and was present in 153 of 1029 tumors without pathogenic germline MMR variants, yielding a sensitivity of 94.4%, specificity of 85.1%, LR+ of 6.35, and LR− of 0.065. Thus, elevated TMB provided moderate‐to‐strong positive evidence for pathogenic germline MMR status, whereas low TMB provided stronger negative evidence than the absence of an MMR‐d mutational signature alone. Comparative performance of whole‐exome MMR‐d signatures, panel‐restricted MMR‐d signature calling, and TMB‐based classification is shown in Figure S4B. Whole‐exome signatures provided informative evidence in both directions, whereas panel‐restricted signatures retained useful positive evidence when strongly positive but generated comparatively weak negative evidence. TMB showed a complementary profile, with a lower LR+ than whole‐exome signatures but a stronger LR−.

## 4. Discussion

In this study, we presented a quantitative Bayesian framework for reclassifying germline VUS in MMR genes via the integration of somatic tumor features. By leveraging tumor mutational signatures, somatic hits, ASE, IHC, and established variant‐level evidence, we demonstrated that somatic tumor biology can be incorporated in a principled and reproducible manner to refine germline variant pathogenicity assessments.

### 4.1. Clinical Concordance and Reclassification Impact

Applied to 45 germline MMR VUS, the framework produced results that were highly concordant with external clinical laboratory reclassifications in subsequent years. Two variants crossed the pathogenic threshold and one reached likely pathogenic, and all three were subsequently or independently classified as likely pathogenic by external laboratories (including Ambry Genetics for both *MLH1* variants), providing orthogonal support for the framework′s conclusions. Among 35 variants downgraded to likely benign, 16 of 18 later downgraded by Invitae were concordant with our Bayesian predictions (Table 4). This agreement supports the framework′s specificity and highlights its potential to reduce persistent VUS burden in routine hereditary cancer genetics workflows.

### 4.2. Impact of Integration Mutational Signatures on Reclassification

Importantly, these reclassifications were not driven by a single feature but, instead, emerged from the cumulative and weighted integration of multiple complementary data modalities. These modalities are not necessarily independent, particularly when they arise from the same underlying MMR‐deficient tumor process. Accordingly, evidence was applied conservatively and was not multiplied when findings were biologically redundant or could not be attributed to the germline VUS gene. In particular, second somatic hits in the same MMR gene as the germline VUS and statistically significant mutant ASE provided mechanistic evidence of biallelic inactivation. When combined with MMR‐deficient mutational signatures, these features shifted posterior probabilities decisively beyond established ACMG thresholds for pathogenicity.

A key contribution of this work is the quantitative calibration of MMR‐deficient mutational signatures as evidence within a Bayesian variant classification framework. Using an empirically derived LR of approximately eight, we demonstrated that the presence of an MMR‐deficient signature increases the probability of an underlying germline pathogenic MMR variant by eightfold in colorectal and ECs. Although this association is not deterministic, given the prevalence of somatic mechanisms such as *MLH1* promoter hypermethylation and double somatic inactivation, it nonetheless provides moderate to strong evidence when considered alongside orthogonal data.

Equally informative was the absence of MMR‐deficient signatures. Germline VUS carriers without evidence of MMR‐deficient mutational processes exhibited low TMB and MSS, leading to consistent downward adjustments of posterior probability. This bidirectional application highlights the utility of mutational signatures not only for upgrading variants but also for confidently downgrading variants toward likely benign classifications.

### 4.3. Operational Decision Rules for IHC and Alternative Explanations

Our decision tree (Figure 1) formalizes how tumor context modifies variant interpretation, particularly when IHC results are available. By distinguishing cases in which protein loss directly implicates the germline VUS gene from those in which loss is explained by alternative somatic mechanisms or complexing partners, we reduce ambiguity and prevent over‐attribution of pathogenicity. This structured approach ensures consistency across cases and enables transparent incorporation of IHC data into Bayesian calculations.

Notably, variants that were upgraded showed IHC patterns concordant with loss of the germline VUS gene and lacked alternative explanations such as promoter hypermethylation. Incorporation of moderate pathogenic evidence from IHC further elevated posterior probabilities for two of the variants, underscoring the value of integrating histopathologic data with genomic analyses.

### 4.4. Resolving Discordant Biomarkers

We specifically addressed the challenge of discordant signals between somatic signatures and IHC. As noted in the literature [50–52], certain missense variants may produce nonfunctional proteins that retain sufficient structural stability to appear intact by IHC, underscoring the importance of interpreting IHC in conjunction with other tumor biomarkers. This was illustrated by the *PMS2* p.Arg578His variant. Although the associated tumor exhibited high TMB and MSI‐high status, it lacked an MMR‐d mutational signature and retained MMR protein expression by IHC. Within our Bayesian framework, the absence of an MMR‐d signature provided benign‐direction evidence (LR − ≈0.156), reducing the posterior probability of pathogenicity to 1.70% and supporting a likely benign classification. The intact IHC findings were concordant with this interpretation despite the elevated TMB and MSI‐high phenotype. This classification was also concordant with subsequent reclassification by Invitae and other clinical laboratories as likely benign. This case illustrates the value of integrating multiple tumor biomarkers rather than allowing elevated TMB or MSI‐high status alone, which may arise through alternative somatic mechanisms, to drive attribution of pathogenicity to a germline VUS. Furthermore, our results highlight the potential of the MMR‐d signature in resolving “attenuated” Lynch syndrome phenotypes, specifically for *MSH6* and *PMS2* variant carriers. It is well‐established that *MSH6*‐deficient tumors will more frequently present with MSI‐low or MSS [53, 54]. In our cohort, we identified two patients harboring pathogenic *MSH6* germline variants with confirmed loss of *MSH6* protein, who were clinically classified as MSS. Despite the absence of the MSI‐High phenotype expected of patients with MMR‐d, our framework detected a robust MMR‐d mutational signature in both cases (Table 2). This underscores a critical advantage of the MMR‐d signature detection, which captures and quantifies the effect of the repair deficiency across the entire exome, compared to MSI‐testing, which interrogates a limited set of loci.

### 4.5. Relationship to ACMG/AMP and Prior Models

Current clinical variant classification largely relies on the ACMG AMP guidelines [19], which provide qualitative categories but limited guidance on how to combine multiple lines of evidence or resolve conflicting signals. Prior multifactorial likelihood models have incorporated family history, segregation, and functional data, but have not treated tumor mutational processes as quantitative evidence.

Our approach extends these frameworks by explicitly mapping diverse somatic data types to LRs calibrated within a Bayesian model. This allows variant interpretation to move beyond categorical assertions toward probabilistic reasoning, offering a continuous measure of confidence that can be updated as new data becomes available. Importantly, this framework does not replace existing clinical interpretation but complements it by providing a transparent and reproducible method for evidence integration.

### 4.6. Translational Implications, Limitations, and Conservative Interpretation

The ability to reclassify MMR VUSs has direct implications for patient management, genetic counseling, and cascade testing in families at risk for Lynch syndrome. By integrating tumor‐derived evidence that is already routinely generated in clinical workflows, this framework enables more informed interpretation without requiring additional experimental assays. The strong concordance with external laboratory reclassifications suggests that such an approach could be operationalized to support clinical decision making and reduce the long‐term burden of unresolved VUSs.

The Bayesian framework proposed here relies on assumptions of independence that may not hold in every clinical scenario; therefore, expert interpretation by physicians and genetic counselors remains essential. In particular, when a patient carries two variants in the same pathway or in close physical proximity, the supporting evidence may be confounded. For example, population prevalence data may be skewed if two VUS are in linkage disequilibrium.

An instructive edge case involved co‐occurring *MSH6* p.Ser1188Asn and *MSH2* p.Val923Glu, a combination previously reported in Lynch syndrome. Although the tumor showed combined loss of *MSH2* and *MSH6* and a strong MMR‐d signature, these phenotypes could not be assigned to a single variant because disruptive C‐terminal *MSH6* variants can destabilize MutS*α* and produce secondary loss of *MSH2*. We therefore excluded shared tumor features from Bayesian updating for both variants and relied on variant‐specific evidence. Under this conservative handling, *MSH6* p.Ser1188Asn reached likely pathogenic based on independent evidence (rarity, functional impairment, and a clonal somatic second hit), whereas *MSH2* p.Val923Glu remained a VUS in the absence of functional deficit [49]. This example illustrates how the framework can operate in complex settings while explicitly recognizing and controlling for confounded tumor phenotypes.

This study has several limitations. First, the LRs were calibrated in a single‐institution cohort restricted to colorectal and ECs, tumor types in which MMR‐d biology is relatively prevalent and well characterized. The performance of MMR‐d signatures, TMB, and related tumor features may differ in other tumor types with distinct background mutational processes, frequencies of sporadic MMR‐d mechanisms, or patterns of germline MMR‐associated tumorigenesis. Extension of this framework beyond colorectal and EC will therefore require tumor‐specific calibration and independent validation.

Second, although the INSPIRE cohort included unselected patients with colorectal and EC rather than only individuals referred for germline testing on the basis of clinical criteria, the variant‐level reclassification analysis included 45 germline MMR VUS. The observed reclassification performance and concordance with subsequent laboratory reassessments are encouraging, but the number of variants available for detailed Bayesian curation remains modest. Larger independent cohorts, including populations with broader ancestral diversity and a wider range of germline variant spectra, will be needed to establish the reproducibility and generalizability of the proposed evidence weights.

Finally, this framework is intended for germline variant interpretation in paired tumor‐normal cases and does not imply that a germline reclassification can be automatically transferred to the same alteration when observed somatically in an unrelated tumor. Somatic interpretation depends on tumor lineage, clonality, co‐occurring alterations, and therapeutic context. Although the general principles described here may be adapted to other cancer predisposition genes with characteristic somatic phenotypes, each application will require disease‐specific and assay‐specific validation.

A practical question is whether targeted panel sequencing can provide mutational‐signature evidence comparable to WES. After panel‐specific trinucleotide renormalization and threshold recalibration, panel‐restricted MMR‐d calls retained a useful LR+, indicating that strongly positive panel‐derived signatures can support pathogenicity. However, panel sensitivity was substantially lower than whole‐exome sensitivity, and panel‐negative calls had LR− values near 0.5, providing little meaningful evidence against pathogenicity. This asymmetry reflects the limited number of SBS events captured within a targeted panel footprint rather than failure of the underlying biological signal. Accordingly, panel‐derived MMR‐d signatures may be useful for ruling in MMR‐d biology when strongly positive, whereas WES remains preferable when negative signature evidence will be used to down‐weight germline pathogenicity.

TMB showed a complementary performance profile. In this cohort, elevated TMB had a lower LR+ than whole‐exome MMR‐d signatures but a stronger LR−, suggesting that low TMB may be particularly informative against a pathogenic germline MMR mechanism. However, TMB and mutational signatures are biologically correlated downstream manifestations of MMR dysfunction. They should therefore not be treated as fully independent evidence sources or multiplied together without an explicitly modeled dependence structure.

### 4.7. Future Directions

Future work should explore prospective validation, integration with additional somatic modalities such as copy number signatures, and automation within clinical reporting systems. As tumor and germline sequencing continue to be performed together, such integrative Bayesian approaches are well positioned to become a cornerstone of variant interpretation.

## Author Contributions

S.W.G., H.H., and D.W.C. have contributed to the work equally and should be regarded as cofirst authors.

## Funding

This work was supported by the National Cancer Institute, 10.13039/100000054, U2C CA252971.

## Conflicts of Interest

The authors declare no conflicts of interest.

## Supporting Information

Additional supporting information can be found online in the Supporting Information section.

## Supporting information


**Supporting Information 1** Figure S1: Somatic variant processing and mutational signature assignment pipeline schematic overview of the somatic variant calling, filtering, and mutational signature assignment workflow used in this study. Tumor and matched germline BAM files aligned to the GRCh38 reference genome were processed using five independent somatic variant callers (Mutect2, Strelka2, Octopus, VarDict, and Lancet). Variant call format (VCF) files from all callers were merged per sample, and only variants detected by at least three of the five callers were retained to reduce false‐positive calls. These consensus variants were written to final per‐sample VCF files and used as input for downstream mutational signature analysis. Mutational signatures were inferred using SigProfilerAssignment (v1.0.21) under the COSMIC v3.6 reference framework in exome mode with SBS96 contexts. An individual signature was considered present when its attributed activity was ≥ 200 mutations. Signature categories included MMR‐d, POLE, POLD1, HRD, MUTYH, APOBEC, tobacco‐associated, and UV‐associated processes, with specimens containing multiple active processes assigned combined classifications. Figure S2: Distribution of pathogenic germline MMR variants and Bayesian impact of MMR‐d signature evidence. (A) Posterior probability curves illustrating the impact of incorporating MMR‐deficiency (MMR‐d) mutational signature evidence on germline variant classification under varying prior probabilities. The likelihood ratio associated with the presence of an MMR‐d signature (LR^+^) was derived from cohort‐level analysis and transformed to log10(LR^+^) to align with ACMG/AMP Bayesian evidence strength thresholds (Supporting = 0.32, Moderate = 0.64, Strong = 1.27, Very Strong = 2.54 [23, 41]). Posterior probabilities were calculated across a range of prior probabilities (0.01–0.80) using Bayes′ theorem to demonstrate how MMR‐d signature evidence shifts variant interpretation. The curve highlights the minimum prior probability required for the addition of MMR‐d signature evidence to reach the “likely pathogenic” classification threshold (posterior probability ≥ 0.90). (B) Pie chart showing the distribution of pathogenic germline MMR gene variants across the study cohort, stratified by affected gene. Percentages reflect the proportion of pathogenic or likely pathogenic variants attributed to each MMR gene among all pathogenic germline cases included in the analysis. Figure S3: Confidence intervals—diagnostic performance and Bayesian evidence strength of the MMR‐d mutational signature. (A) Sensitivity and specificity (points) with 95% confidence intervals (horizontal whiskers) derived from the confusion matrix in Figure 4B. The vertical dashed line marks a reference proportion of 0.5. (B) Likelihood ratios with 95% confidence intervals. LR+ summarizes evidence supporting germline pathogenicity when an MMR‐d signature is present; the negative‐evidence term is shown as (1 − sensitivity)/specificity, LR − = 0.156. The vertical dashed line marks LR = 1 (no evidence). (C) Evidence scale shown as log10‐transformed likelihood ratios with 95% confidence intervals; the vertical dashed line marks log10(LR) = 0 (no evidence). Points denote point estimates and whiskers denote 95% confidence intervals. Figure S4: Performance of panel‐restricted mutational signature analysis relative to whole‐exome signatures and TMB. (A) Calibration of panel‐restricted MMR‐d signature calling across summed activity thresholds for SBS6, SBS14, SBS15, SBS20, SBS21, SBS26, and SBS44. Sensitivity, specificity, and Youden′s J statistic are shown across candidate thresholds. The threshold of k ≥ 5 maximized Youden′s J and was selected as the panel operating point with the greatest overall diagnostic performance. A more stringent threshold of *k* ≥ 8 was selected as an alternative operating point that produced an LR+ comparable to the whole‐exome analysis while retaining sensitivity above 50%.(B) Sensitivity‐specificity tradeoff for tumor‐level indicators of pathogenic germline MMR status. Points represent whole‐exome MMR‐d signature status, panel‐restricted MMR‐d signature status at *k* ≥ 5 and *k* ≥ 8, and TMB of at least 10 mut/Mb. Whole‐exome signatures provided higher sensitivity than panel‐restricted signatures, whereas the *k* ≥ 8 panel threshold achieved higher specificity at the cost of reduced sensitivity. TMB demonstrated the highest sensitivity but lower specificity than signature‐based approaches.

**Supporting Information 2** Table S1 Clinical, demographic, and genomic features of 110 patients with MMR‐d mutational signatures in the absence of a known pathogenic germline MMR variant. Shown are clinical, demographic, and molecular features for 110 tumor specimens from 107 unique patients whose tumors exhibited MMR‐d mutational signatures despite the absence of a known pathogenic germline MMR variant. Each row represents an individual tumor specimen. Data include specimen site, TMB (reported as mutations per megabase), MSI status derived from tumor DNA, and the dominant COSMIC mutational signature identified through WES. Somatic variants in MMR genes (*MLH1*, *MSH2*, *MSH6*, and *PMS2*) are listed alongside their respective allele frequencies; samples were flagged as having a somatic MMR “hit” if at least one high‐confidence somatic mutation was present. Self‐reported demographic data include sex, race, ethnicity, and age at diagnosis. The diagnostic classification (“Diag Call”) reflects manual review of each case to determine whether the observed MMR‐d signature is likely a FP in the context of no germline explanation. Cancer type is shown in the final column.

**Supporting Information 3** Table S2: Clinical, molecular, and mutational features of patients with pathogenic or likely pathogenic germline MMR variants. Shown are clinical, molecular, and mutational features of 35 patients harboring pathogenic or likely pathogenic germline variants in MMR genes identified through clinical germline testing. Tumors were evaluated for evidence of MMR‐d, including COSMIC mutational signatures, TMB, and MSI. The diagnostic call reflects concordance between the germline pathogenic variant and the tumor phenotype: cases labeled as “TP” (true positive) exhibited MMR‐d signatures consistent with the underlying germline finding, whereas cases labeled as “FN” (false negative) lacked somatic evidence of MMR‐d. Each row includes the patient′s cancer type (colorectal or endometrial), gene affected, Invitae classification at the time of sequencing and genomic coordinates following GRCh37 HGVS nomenclature. Tumor site, numerical TMB score (in mutations per megabase), TMB tier, MSI status, sex, and age at diagnosis are also provided. This table expands on the cohort summarized in Table 2 of the main manuscript.

**Supporting Information 4** Table S3: Clinical, molecular, and mutational features of patients with germline MMR VUS. Shown are patients with germline MMR variants initially classified as VUS based on Invitae reports at the time of sequencing. The table includes tumor molecular characteristics such as MSI status, TMB, and dominant COSMIC mutational signatures. Columns also report the presence or absence of secondary somatic mutations in MMR genes (*MLH1*, *MSH2*, *MSH6*, and *PMS2*), with corresponding allele frequencies (AFs). Diagnostic calls reflect whether the somatic and molecular findings are consistent with a MMR‐d phenotype, with FP indicating false positives (MMR‐d signature present without a confirmed pathogenic germline variant), and TN indicating true negatives (no MMR‐d features and no germline pathogenic variant). Variant classifications from both Invitae and ClinVar are listed, along with HGVS nomenclature based on GRCh37. Some patients have multiple tumor sites or multiple VUSs in the same gene.

## Data Availability

The data that support the findings of this study are available on request from the corresponding author. The data are not publicly available due to privacy or ethical restrictions.
