# Supplementary material for: Bayesian Integration of Tumor Mutational Signatures and Somatic Features Refines Pathogenicity Assessment of Germline Mismatch Repair Variants
Source: Hum Mutat. 2026 Sep 8;2026:7456837. doi: 10.1155/humu/7456837 (PMC13554728; doi:10.1155/humu/7456837)
Supplement: Supplementary file 1 [file HUMU-2026-7456837-s003.docx]

**SUPPLEMENTAL FIGURES**

**Bayesian Integration of Tumor Mutational Signatures and Somatic Features Refines Pathogenicity Assessment of Germline Mismatch Repair Variants**

Yonatan Amzaleg^1^, Kevin McDonnell^2^, Gregory Idos^3^, Christina Mathai^4^, Stacy W. Gray^†,2,3^, Heather Hampel^†,2,5^, David W Craig ^†,1,5^

^†^ Co-senior authors

^1^ Department of Integrative Translational Science, Beckman Research Institute, City of Hope, Duarte, CA, USA.

^2^ Division of Clinical Cancer Genomics, Department of Medical Oncology & Therapeutics Research, City of Hope, Duarte, CA, USA.

^3^ Department of Population Sciences, City of Hope Comprehensive Cancer Center, Duarte, CA, USA.

^4^ Enterprise Data Group, City of Hope, Duarte, CA, USA.

^5^ Corresponding: David Craig (dacraig@coh.org) and Heather Hampel ([Hhampel@coh.org](mailto:Hhampel@coh.org))

**Supplemental Figure S1. Somatic Variant Processing and Mutational Signature Assignment Pipeline** Schematic overview of the somatic variant calling, filtering, and mutational signature assignment workflow used in this study. Tumor and matched germline BAM files aligned to the GRCh38 reference genome were processed using five independent somatic variant callers (Mutect2, Strelka2, Octopus, VarDict, and Lancet). Variant call format (VCF) files from all callers were merged per sample, and only variants detected by at least three of the five callers were retained to reduce false-positive calls. These consensus variants were written to final per-sample VCF files and used as input for downstream mutational signature analysis. Mutational signatures were inferred using SigProfilerAssignment under the COSMIC v3.4 reference framework in exome mode with 96-channel single base substitution (SBS) contexts. Samples with at least 200 SBS mutations were retained for analysis. Signature classification was based on the dominant COSMIC SBS activities observed, including mismatch repair deficiency (MMR-d), POLE, POLD1, homologous recombination deficiency (HRD), MUTYH, APOBEC, smoking-related, and ultraviolet-associated signatures, as indicated.

**Supplemental Figure S2. Distribution of Pathogenic Germline MMR Variants and Bayesian Impact of MMR-d Signature Evidence** **(A)** Posterior probability curves illustrating the impact of incorporating MMR-deficiency (MMR-d) mutational signature evidence on germline variant classification under varying prior probabilities. The likelihood ratio associated with the presence of an MMR-d signature (LR⁺) was derived from cohort-level analysis and transformed to log10(LR⁺) to align with ACMG/AMP Bayesian evidence strength thresholds (Supporting = 0.32, Moderate = 0.64, Strong = 1.27, Very Strong = 2.54^23,51^). Posterior probabilities were calculated across a range of prior probabilities (0.01–0.80) using Bayes’ theorem to demonstrate how MMR-d signature evidence shifts variant interpretation. The curve highlights the minimum prior probability required for the addition of MMR-d signature evidence to reach the “likely pathogenic” classification threshold (posterior probability ≥0.90).**(B)** Pie chart showing the distribution of pathogenic germline mismatch repair (MMR) gene variants across the study cohort, stratified by affected gene. Percentages reflect the proportion of pathogenic or likely pathogenic variants attributed to each MMR gene among all pathogenic germline cases included in the analysis. were calculated across a range of prior probabilities (0.01–0.80) using Bayes’ theorem to demonstrate how MMR-d signature evidence shifts variant interpretation. The curve highlights the minimum prior probability required for the addition of MMR-d signature evidence to reach the “likely pathogenic” classification threshold (posterior probability ≥0.90).

**Supplemental Figure S3. Confidence Intervals - Diagnostic performance and Bayesian evidence strength of the MMR-d mutational signature**. (A) Sensitivity and specificity (points) with 95% confidence intervals (horizontal whiskers) derived from the confusion matrix in Figure 2B. The vertical dashed line marks a reference proportion of 0.5. **(B)** Likelihood ratios with 95% confidence intervals. LR+ summarizes evidence supporting germline pathogenicity when an MMR-d signature is present; the negative-evidence term is shown as 1-(Sensitivity)/Specificity. The vertical dashed line marks LR = 1 (no evidence). **(C)** Evidence scale shown as log10-transformed likelihood ratios with 95% confidence intervals; the vertical dashed line marks log10(LR) = 0 (no evidence). Points denote point estimates and whiskers denote 95% confidence intervals.

**s**

**Supplemental Figure S4. Performance of panel-restricted mutational signature analysis relative to whole-exome signatures and tumor mutational burden. (A)** Calibration of panel-restricted MMR-d signature calling across summed activity thresholds for SBS6, SBS14, SBS15, SBS20, SBS21, SBS26, and SBS44. Sensitivity, specificity, and Youden’s J statistic are shown across candidate thresholds. The threshold of k ≥ 5 maximized Youden’s J and was selected as the panel operating point with the greatest overall diagnostic performance. A more stringent threshold of k ≥ 8 was selected as an alternative operating point that produced an LR+ comparable to the whole-exome analysis while retaining sensitivity above 50%.**(B)** Sensitivity-specificity tradeoff for tumor-level indicators of pathogenic germline MMR status. Points represent whole-exome MMR-d signature status, panel-restricted MMR-d signature status at k ≥ 5 and k ≥ 8, and tumor mutational burden of at least 10 mutations/Mb. Whole-exome signatures provided higher sensitivity than panel-restricted signatures, whereas the k ≥ 8 panel threshold achieved higher specificity at the cost of reduced sensitivity. TMB demonstrated the highest sensitivity but lower specificity than signature-based approaches.

**SUPPLEMENTAL TABLE LEGENDS (XLS)**

**Supplemental Table 1** **Clinical, demographic, and genomic features of 111 patients with mismatch repair–deficient (MMR-d) mutational signatures in the absence of a known pathogenic germline MMR variant.** Shown are clinical, demographic, and molecular features for 110 patients whose tumors exhibited mismatch repair–deficient (MMR-d) mutational signatures despite the absence of a known pathogenic germline MMR variant. Each row represents an individual patient case. Data include specimen site, tumor mutational burden (TMB, reported as mutations per megabase), MSI status derived from tumor DNA, and the dominant COSMIC mutational signature identified through whole-exome sequencing. Somatic variants in MMR genes (*MLH1*, *MSH2*, *MSH6*, *PMS2*) are listed alongside their respective allele frequencies; samples were flagged as having a somatic MMR “hit” if at least one high-confidence somatic mutation was present. Self-reported demographic data include gender, race, ethnicity, and age at diagnosis. The diagnostic classification (“Diag Call”) reflects manual review of each case to determine whether the observed MMR-d signature is likely a false positive in the context of no germline explanation. Cancer type is shown in the final column.

**Supplementary Table 2 Clinical, molecular, and mutational features of patients with pathogenic or likely pathogenic germline MMR variants** Shown are clinical, molecular, and mutational features of 35 patients harboring pathogenic or likely pathogenic germline variants in mismatch repair (MMR) genes identified through clinical germline testing. Tumors were evaluated for evidence of MMR deficiency, including COSMIC mutational signatures, tumor mutational burden (TMB), and microsatellite instability (MSI). The diagnostic call reflects concordance between the germline pathogenic variant and the tumor phenotype: cases labeled as “TP” (true positive) exhibited MMR-d signatures consistent with the underlying germline finding, whereas cases labeled as “FN” (false negative) lacked somatic evidence of MMR deficiency. Each row includes the patient’s cancer type (colorectal or endometrial), gene affected, Invitae classification at the time of sequencing and genomic coordinates following GRCh38 HGVS nomenclature. Tumor site, numerical TMB score (in mutations per megabase), TMB tier, MSI status, gender, and age at diagnosis are also provided. This table expands on the cohort summarized in Table 1 of the main manuscript.

**Supplementary Table 3. Clinical, molecular, and mutational features of patients with germline MMR variants of uncertain significance (VUS)** Shown are patients with germline MMR variants initially classified as variants of uncertain significance (VUS) based on Invitae reports at the time of sequencing. The table includes tumor molecular characteristics such as microsatellite instability (MSI) status, tumor mutational burden (TMB), and dominant COSMIC mutational signatures. Columns also report the presence or absence of secondary somatic mutations in mismatch repair genes (*MLH1*, *MSH2*, *MSH6*, *PMS2*), with corresponding allele frequencies (AFs). Diagnostic calls reflect whether the somatic and molecular findings are consistent with a mismatch repair-deficient phenotype, with FP indicating false positives (MMR-d signature present without confirmed pathogenic germline variant), and TN indicating true negatives (no MMR-d features and no germline pathogenic variant). Variant classifications from both Invitae and ClinVar are listed, along with HGVS nomenclature based on GRCh38. Some patients have multiple tumor sites or multiple VUSs in the same gene.
